# Overexpression of *LcSABP*, an Orthologous Gene for Salicylic Acid Binding Protein 2, Enhances Drought Stress Tolerance in Transgenic Tobacco

**DOI:** 10.3389/fpls.2019.00200

**Published:** 2019-02-21

**Authors:** Qian Li, Gang Wang, Chunfeng Guan, Dan Yang, Yurong Wang, Yue Zhang, Jing Ji, Chao Jin, Ting An

**Affiliations:** ^1^School of Environmental Science and Engineering, Tianjin University, Tianjin, China; ^2^Division of Biological Sciences, University of California, San Diego, San Diego, CA, United States

**Keywords:** salicylic acid, *LcSABP*, drought stress, tobacco, antioxidant enzyme, photosynthetic capacity, reactive oxygen species, stress-responsive genes

## Abstract

Salicylic acid (SA) plays an essential role in the growth and development of plants, and in their response to abiotic stress. Previous studies have mostly focused on the effects of exogenously applied SA on the physiological response of plants to abiotic stresses; however, the underlying genetic mechanisms for the regulatory functions of endogenous SA in the defense response of plants remain unclear. In plants, SA binding protein 2 (SABP2), possessing methyl salicylate (MeSA) esterase activity, catalyzes the conversion of MeSA to SA. Herein, a *SABP2*-like gene, *LcSABP*, was cloned from *Lycium chinense*, which contained a complete open reading frame of 795 bp and encoded a protein of 264 amino acids that shared high sequence similarities with SABP2 orthologs from other plants. Overexpression of *LcSABP* enhanced the drought tolerance of transgenic tobacco plants. The results indicated that increased levels of *LcSABP* transcripts and endogenous SA content were involved in the enhanced drought tolerance. Physiological and biochemical studies further demonstrated that higher chlorophyll content, increased photosynthetic capacity, lower malondialdehyde content, and higher activities of superoxide dismutase, peroxidase, and catalase enhanced the drought tolerance of transgenic plants. Moreover, overexpression of *LcSABP* also increased the expression of reactive oxygen species (ROS)- and stress-responsive genes under drought stress. Overall, our results demonstrate that *LcSABP* plays a positive regulatory role in drought stress response by enhancing the endogenous SA content, promoting the scavenging of ROS, and regulating of the expression of stress-related transcription factor genes. Our findings indicate that *LcSABP* functions as a major regulator of the plant’s response to drought stress through a SA-dependent defense pathway.

## Introduction

Abiotic stresses, such as drought and high salt stress, are thought to be the main factors adversely affecting plant growth and crop productivity, which limit global agricultural production (An et al., 2015; Wei T. et al., 2017). The adaptation of plants to environmental stress involves numerous biochemical reactions and physiological processes (Cramer et al., 2011; Thomas, 2015). A number of genes and their proteins respond to stresses at transcriptional and translational levels, functioning as hormone signaling molecules and TFs that regulate signal transduction and gene expression during stress response (Sreenivasulu et al., 2007; Wei et al., 2016). SA is a naturally occurring phenolic compound and is one of the most important signaling molecules involved in the regulation of plant development, maturation, aging, and other physiological processes (Vlot et al., 2009; Klessig et al., 2018).

Salicylic acid is considered to be one of the key signals in plant defense mechanisms, and its relationship with plant stress resistance has been the focus of several studies. It is clear that SA can be used as a signaling molecule to induce plant disease resistance by activating the plant defense system (Lin et al., 2016; Klessig et al., 2018). It also plays important roles in tolerance to abiotic stresses, such as those induced by salt, heat, and heavy metals (Kang et al., 2014). Exogenous application of SA to ASD16 (salt-sensitive) and BR26 (salt-tolerant) varieties of rice ameliorated the decrease in the rate of germination, growth, and yield caused by salt stress (Jini and Joseph, 2017). Foliar application of SA improved the adverse effects of lead stress on the growth of pea plants (Ghani, 2015). Treatment of SA also increased the RWC of barley leaves under salt stress (Kovacik et al., 2009). The application of SA significantly enhanced the tolerance of naked oat plants to salt stress, which might be associated with hydrogen peroxide homeostasis in plant cells (Xu et al., 2008). The relationship between SA and ROS is complex. Horváth et al. (2007) reported that SA can scavenge active oxygen directly or by regulating the activity of antioxidant enzymes; low concentration of SA was reported to increase the activity of such enzymes (Ashraf et al., 2010). Although SA was shown to induce plants’ response to abiotic stress (Panda and Patra, 2007; Kang et al., 2012), most of the previous studies have focused on deciphering the mechanism of modulation of stress response by exogenous SA (Zhang et al., 2011), and the roles of endogenous SA remain poorly understood (Asensi-Fabado and Munné-Bosch, 2011; Miura et al., 2013).

In recent years, the biosynthetic and metabolic pathways of SA have been elucidated (Vlot et al., 2009; Dempsey et al., 2011). It has been suggested that SA is synthesized via two different pathways, namely the isochorismate pathway and the phenylalanine ammonia-lyase pathway. Several key enzymes, such as benzoic acid 2-hydroxylase (BA2H), isochorismate pyruvate lyase (IPL), phenylalanine ammonia lyase (PAL), isochorismate synthase (ICS), SA glucosyltransferase (SAGT), SA carboxyl methyltransferase (SAMT), and SA binding protein (SABP), catalyzing the conversion of intermediates in SA biosynthesis pathway, have been shown to play key roles in the biosynthesis and metabolism of SA (Dempsey et al., 2011; Miura and Tada, 2014).

Presently, four types of enzymatically active SABPs are identified, which function as chloroplastic carbonic anhydrase, CAT, cytoplasmic ascorbate APX, or MeSA esterase (Du and Klessig, 1997; Slaymaker et al., 2002; Kumar and Klessig, 2003; Vlot et al., 2008). In tobacco plants, SABP2, which possesses esterase activity, was shown to catalyze the demethylation of inactive MeSA, transported to distal tissues through phloem, to the active SA (Chen and Klessig, 1991; Kumar and Klessig, 2003). SABPs have been identified and characterized from many plant species (Tripathi et al., 2010; Kohli et al., 2017). A gene family from *Arabidopsis thaliana* encoding 18 potentially active a/b fold hydrolases, sharing 32–57% identity with SABP2, was characterized. Among these, five members showed MeSA esterase activity. Moreover, conditional expression of AtMES1, -7, and -9 complemented the systemic acquired resistance (SAR) deficiency in SABP2-silenced tobacco, indicating that they were functional homologs of SABP2 (Vlot et al., 2008). The enzymatic activity of SABP2, which hydrolyzes MeSA to SA, was required for successful resistance of tobacco systemically infected with tobacco mosaic virus (Park et al., 2007; Vlot et al., 2008). A soluble SA-binding protein was detected in tobacco leaves and was partly analyzed; this protein was predicted to perceive and transduce the SA signal to corresponding components, which ultimately activated a series of positive responses to disease resistance in plants (Chen and Klessig, 1991; Fritig and Legrand, 1993). In tobacco, SABP2 is localized to the cell membrane and possesses APX (APX) and MeSA esterase activities (Du and Klessig, 1997; Forouhar et al., 2005). In a previous study, the presence of an SA-binding protein (SABP3), identified as a chloroplast carbonic anhydrase (CA), was reported in the soluble fraction of purified chloroplasts from tobacco leaf, which might also play important roles in allergic reactions owing to its antioxidant capacity (Slaymaker et al., 2002). In *A. thaliana*, the CA activity of chloroplastic carbonic anhydrase1 (AtCA1 or AtSABP3) and its binding to SA are essential to restrict the spread and accumulation of virus, via the induction of SA accumulation and triggering of the SA pathway (Wang et al., 2009; Poque et al., 2018). To better understand the varied functions of SA, particularly in activating disease resistance, and to identify candidate SABPs (cSABPs) in *Arabidopsis*, two high-throughput strategies were developed using biochemical and biophysical methods. In these two high-throughput screens, nine new and more than 100 candidate SABPs were identified (Manohar et al., 2014). The above-mentioned reports mainly focused on structural characteristics of SABP and on the regulation of the response of plants to biotic stress by SABP. However, research on the relationship between SABP and abiotic stress has been lacking, and the roles of *SABP* gene in the response of plants to abiotic stress remain unknown.

*Lycium chinense* is a deciduous dicotyledonous shrub. It is a highly tolerant plant that can grow in arid, cold, and salty environment. The functional analysis of *SABP* from *L. chinense* has not been done so far. To better identify the roles of *SABP* in drought stress tolerance with the aim of providing a potential genetic resource for improvement of drought resistance in plants, a *SABP2*-like gene, *LcSABP*, was isolated from *L. chinense* and functionally analyzed in the present study. Furthermore, *LcSABP*-overexpressing tobacco plants were obtained via *Agrobacterium*-mediated genetic transformation. The effects of *LcSABP* overexpression on drought tolerance of tobacco plants were assessed by investigating the endogenous SA content, photosynthetic system, antioxidant enzyme activities, and changes in the expression of ROS-related and stress-responsive TFs genes in transgenic plants under drought stress. Our study demonstrates that a *SABP2* ortholog, *LcSABP*, isolated from *L. chinense* is involved in the defense pathway against drought in plants.

## Materials and Methods

### Vector Construction

The full-length open reading frame of *LcSABP* (GenBank accession number: MH598522) was PCR-amplified from *L. chinense* cDNA using LcSABP-F1/R1 primers (Table 1). The p35S::LcSABP plasmid was constructed by ligating *LcSABP* PCR product and plant binary vector pCAMBIA2300, which contained the CaMV35S constitutive promoter (Figure 3A). The p35S::LcSABP plasmid was transformed into *Agrobacterium tumefaciens* strain EHA105 using the standard heat shock method.

**Table 1:** Sequences of specific primers in *LcSABP* cloning, PCR and RT-PCR detection.

Description	Forward (5′–3′)	Reverse (5′–3′)
LcSABP-F1/R1	ATGGCGACTATTGAGAAAGAAGG	TCAGTTGTATTTATGGGCAATCTCC
LcSABP-F2/R2	ATGGCGACTATTGAGAAAGAAGG	GTAGAAATCAGTTGTATTTATGGGC


### Bioinformatics Analysis of the *LcSABP* Gene

The *LcSABP* cDNA was analyzed by BLAST algorithm1. Multiple amino acid sequence alignment analysis was executed using the sequences of LcSABP and other SABP2 orthologs from different plant species obtained from NCBI database using the DNAMAN software. The phylogenetic tree was constructed by the neighbor-joining method using MEGA (version 6.0). The theoretical molecular weight and isoelectric point were calculated using the ProtParam tool.

### Plant Materials, Genetic Transformation, and Molecular Characterization

*Nicotiana tabacum* seeds were surface sterilized with 0.5% NaClO and germinated on MS agar before being used for transformation. The seeds were incubated in an incubator at 28°C under 16/8 h (light/dark) photoperiod. The tobacco plants were transformed by *Agrobacterium*-mediated leaf-disk method, and then the leaf disks were transferred to fresh MS medium. Kanamycin (100 mg/L) was used for selection and was replenished every 10 days. Adventitious shoots were excised and transferred to the hormone-free MS medium supplemented with 100 mg/L kanamycin until roots were induced from the regenerated stems (Zheng et al., 2015). The rooted plantlets were transferred to new MS basal medium for propagation. The 1-month-old seedlings were selected for evaluation using molecular biology techniques, such as PCR. The positive transgenic plants were transferred to aseptic nutrient soil for cultivation, and then the plants showing similar state of growth were selected for the stress treatment experiment.

To verify the presence of transgene, putative T2 homozygous transgenic tobacco plants were screened preliminarily by PCR amplification of the *LcSABP* gene. The kanamycin-resistant plants were selected and total genomic DNA was extracted using the modified cetyltrimethylammonium bromide (CTAB) extraction method as previously described (Zheng et al., 2015). The positive transgenic lines were detected by PCR amplification of *LcSABP* gene fragments using the LcSABP-F1/R1 primer pair. The sequences of primers are listed in Table 1.

Total RNA from WT and transgenic plants was extracted using TRIzol reagent (Invitrogen, Carlsbad, CA, United States). The cDNA was synthesized as per the manufacturer’s instructions (Takara, Japan). To determine the expression levels of *LcSABP* in transgenic tobacco plants, semi-quantitative PCR (RT-PCR) was performed using the LcSABP-F1/R1 primers (Table 1). The *NtActin* gene was used as an internal control.

To evaluate the expression levels of *LcSABP* in transgenic tobacco plants under normal conditions and expression pattern of *LcSABP* in *L. chinense* under drought stress treatment, quantitative real-time PCR (qRT-PCR) was performed using the LcSABP-F2/R2 primers (Table 1) in a Real-time System (qTOWER Applied Biosystems, Jena, Germany) with SYBR Green PCR Master Mix (Takara, Japan). The relative expression level of *LcSABP* was calculated using the 2^-ΔΔCt^ method. The *LcActin* gene was used as an internal control. The amplification for each sample was done in three biological replicates.

### Stress Treatment

For drought stress treatment, the *L. chinense* seedlings were carefully rinsed with 1/2 MS and transferred to 1/2 MS solution containing 20% PEG6000. All the samples were collected at the indicated time points (0, 2, 6, 12, 24, and 72 h), immediately frozen in liquid nitrogen, and stored at -80°C for further analysis.

To test the drought tolerance of transgenic tobacco plants, transgenic and WT tobacco plants showing similar growth were selected and transferred to pots filled with sterile nutrient soil and grown at 28°C under a 16/8 h (light/dark) photoperiod. Before the drought treatment, the pots were placed in trays filled with water overnight and were subsequently moved to dry trays. After 14 days of drought stress, a total of four lines in both the transgenic and WT tobacco plants were selected, with three plants from each line taken as three biological replicates. The third leaf of each plant was used for determination of physiological and biochemical indicators, such as RWC. All the plants showed severe symptoms of wilting after 20 days of drought stress treatment, and then the plants were re-watered.

### Determining of Physiological Indices

The RWC and chlorophyll content of all plants were determined as described in previous studies (Lin et al., 2008; Reis et al., 2014). The RWC of tobacco leaves was determined using dry weight method (Sun et al., 2015). The leaf gas exchange parameters, including stomatal conductance (*g*_s_), transpiration rate (*E*), and net photosynthesis rate (*P*_n_) were measured using a portable gas exchange system (LI-6400XT). The H_2_O_2_ and O_2_^-^ levels, MDA content, proline content, activities of antioxidant enzymes, including SOD, APX, and CAT were evaluated by enzyme-linked immunosorbent assay using detection kits (DRE-P0811c, DRE-P2373c, DRE-P0612c, DRE-P0851c, DRE-P3396c, DRE-P3390c, DRE-P3327c, Kamai Shu, Shanghai, China), according to the manufacturer’s instructions. Each sample had three biological replicates.

### qRT-PCR Analysis of the Genes Regulated in Transgenic *N. tabacum* Under Drought Stress

For qRT-PCR analysis of the genes regulated in transgenic tobacco plants under drought stress, the leaf samples were collected before and after drought stress treatment, as described above. RNA extraction and cDNA synthesis were also performed as mentioned above. The transcriptional levels of the eleven genes encoding the enzyme involved in ABA biosynthesis 9-*cis*-epoxycarotenoid dioxygenase NCED (NtNCED1), the TFs related to stress, such as dehydration responsive element binding protein (NtDREB3), transcription factor Myb1 (NtMYB1), WRKY transcription factor 8 (NtWRKY8), dehydration-responsive protein RD22-like (NtRD22), zinc finger (C2H2 type) transcription factor ZAT10 (NtZAT10), zinc finger protein ZAT12 (NtZAT12), heat shock transcription factor HSFA2 (NtHSFA2), as well as antioxidant enzymes (NtSOD, NtCAT, and NtAPX) were examined. The *NtActin* gene was used as the internal control. The sequences of primers used for qRT-PCR are listed in Table 2. All the assays for each gene were performed in triplicate synchronously under identical conditions.

**Table 2:** Sequences of primers in qRT-PCR.

Description	Forward (5′–3′)	Reverse (5′–3′)
*NtDREB3*	GCCGGAATACACAGGAGAAG	CCAATTTGGGAACACTGAGG
*NtWRKY8*	GTCCATTTCATTTGGGGATG	TTCCATCTTTTTGGGTCTGG
*NtMYB1*	GACTGCGGTGGACGAATTAT	TGCTGCTATTGCAGACCATC
*NtSOD*	CTCCTACCGTCGCCAAAT	GCCCAACCAAGAGAACCC
*NtCAT*	AGGTACCGCTCATTCACACC	AAGCAAGCTTTTGACCCAGA
*NtAPX*	GCTGGAGTTGTTGCTGTTGA	TGGTCAGAACCCTTGGTAGC
*NtRD22*	GCTGTAGTTTGCCACAAGCA	AGCCTTTGTTCCATCAGCAC
*NtZAT10*	AGGAGGAGATGACCAGTCCA	CTCGTGAGTTTTTCCGCTTC
*NtZAT12*	CACCGTGCAAGTCATAAACG	CACCTAAGGCTTGACCCAAA
*NtHSFA2*	GTTCTCTGCTGCATTGGACA	CTTCACCGAGCAACTCTTCC
*NtNCED1*	AAGAATGGCTCCGCAAGTTA	GCCTAGCAATTCCAGAGTGG
*NtActin*	GGAAACATAGTGCTCAGTGGTG	GCTGAGGGAAGCCAAGATAG
*LcActin*	GGGAATTGCTGATAGAATG	AGGGAAGCCAAGATAGAG


### Extraction and Quantification of Endogenous, SA and MeSA

The total endogenous SA and MeSA in tobacco leaves were extracted according to the method described in previous reports (Molders et al., 1996; Seskar et al., 1998; Siegrist et al., 2000). The quantification of the total SA content in plant samples was done with a plant SA ELISA test kit (DRE-P0874c, Kamai Shu, Shanghai, China), as described by the manufacturer. MeSA was extracted as previously described (Engelberth et al., 2003; Song et al., 2008). The quantification of endogenous MeSA was done using the plant salicylate methyl ester ELISA test kit (DRE-P0805b, Kamai Shu, Shanghai, China) as per the manufacturer’s instructions. Each experiment was repeated three times for three individual lines with three biological replicates.

### Determination of ABA Content and Stomatal Aperture

For ABA quantification, ABA in tobacco leaves was extracted as described in previous studies (Huiru et al., 2014; Ijaz et al., 2017), and the endogenous ABA content was measured using an ELISA Kit (DRE-P0800c, Kamai Shu, Shanghai, China) according to the manufacturer’s instructions.

To assess the stomatal response, the stomatal apertures from the leaves of transgenic and WT plants after drought stress were performed as previously described (Huiru et al., 2014; Chu et al., 2015). The ratio of stomatal width to length indicated the degree of stomatal closure. At least 20 stomatal apertures were measured for each line.

### Statistical Analysis

All data were obtained for at least three replicates, each of which contained three biological replicates. Data are presented as means ± standard deviation (SD) and were analyzed using SPSS statistical software (ver.18.0, SPSS Inc., Chicago, IL, United States). Tukey’s test (*P* < 0.05) was applied for determining statistical significance of the differences between WT and transgenic plants.

## Results

### Cloning and Bioinformatics Analysis of *LcSABP*

**FIGURE 1:**
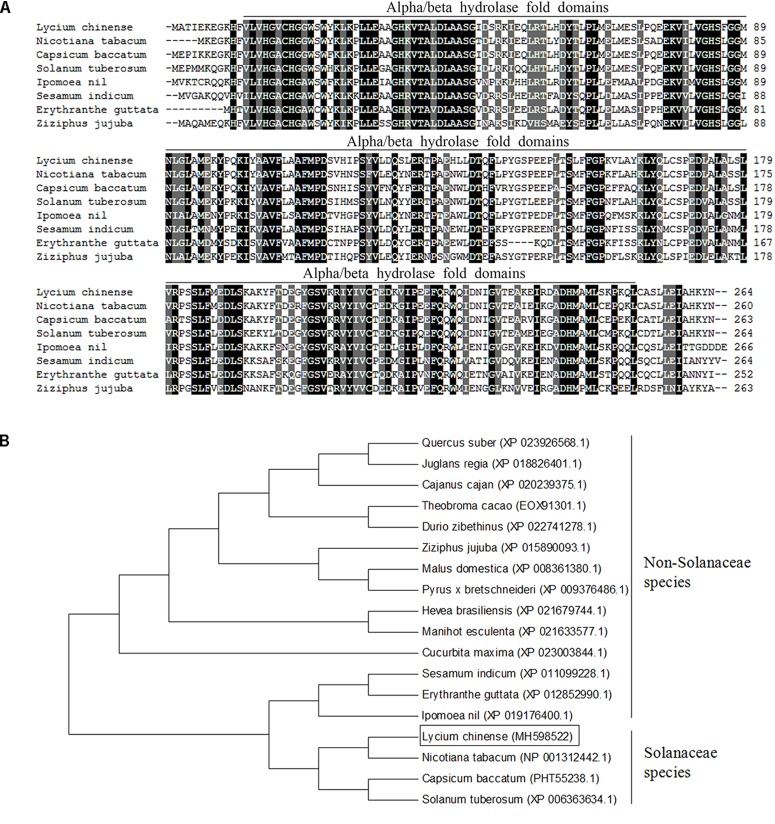
Bioinformatics analysis of the LcSABP protein. **(A)** Multiple sequence alignment of LcSABP with its homologous proteins from other plant species. Identical amino acids are shaded in black, and similar amino acids are shaded in gray. **(B)** Phylogenetic tree of LcSABP with its homologous proteins from other plant species. The phylogenetic tree was created with the neighbor-joining method using the MEGA 6.0 software. The numbers above or below the branches indicate the bootstrap values from 1000 replicates. LcSABP is boxed.

We isolated a *SABP2*-like gene by RT-PCR from *L. chinense* and designated it as *LcSABP* (GenBank Accession: MH598522). The sequence analysis showed that the open reading frame of *LcSABP* was 795-bp long and encoded a deduced protein containing 264 amino acids. The predicted molecular weight and isoelectric point of *LcSABP* were 29.75 kDa and 5.78, respectively. The deduced LcSABP sequence contained the conserved alpha/beta hydrolase folds typical of esterase enzymes. The results of multiple sequence alignment indicated that LcSABP shared high sequence identity with other SABP2 proteins from *N. tabacum* (NP_001312442.1, 88%), *Capsicum baccatum* (PHT55238.1, 84%), *Solanum tuberosum* (XP_006363634.1, 83%), *Ipomoea nil* (XP_019176400.1, 71%), *Sesamum indicum* (XP_011099228.1, 66%), *Erythranthe guttata* (XP_012852990.1, 67%), and *Ziziphus jujuba* (XP_015890093.1, 63%) (Figure 1A). From the phylogenetic data, it is evident that LcSABP had a closer relationship with SABP2 from *N. tabacum* than with those from other plant species (Figure 1B).

### Expression Pattern of *LcSABP* in *L. chinense* Plants

**FIGURE 2:**
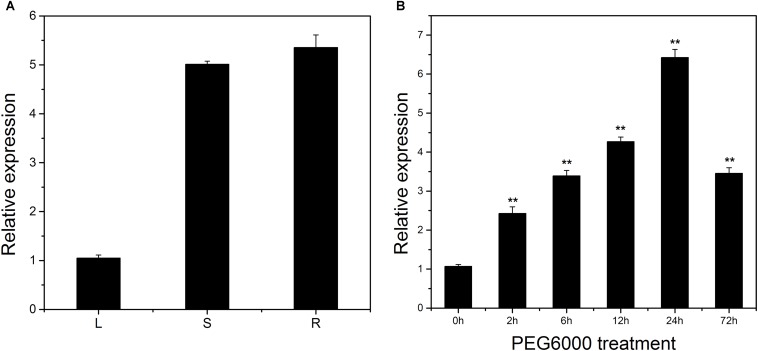
Organ expression assay of *LcSABP* in *Lycium chinense* and expression level of *LcSABP* under treatment with PEG6000. **(A)** Organ expression assay of *LcSABP* in *L. chinense*. The organs (leaf, stem, and root) are represented by L, S, and R, respectively. **(B)** 20% PEG6000 treatment. Bars represent the mean ± SE of three independent experiments. ^∗∗^Significantly different at the *P* < 0.01 level.

The expression pattern of *LcSABP* in different tissues of *L. chinense* was determined using qRT-PCR. *LcSABP* was expressed in all the three tissues (leaf, stem, and root) that were analyzed, with the expression in root being higher compared to that in leaf and stem (Figure 2A). The transcript of *LcSABP* began to accumulate 2 h after PEG6000 treatment and reached maximum at 24 h, which was 5.99-fold of the initial level, followed by a decrease at the end of the treatment (Figure 2B).

### Generation of Transgenic Tobacco Plants

**FIGURE 3:**
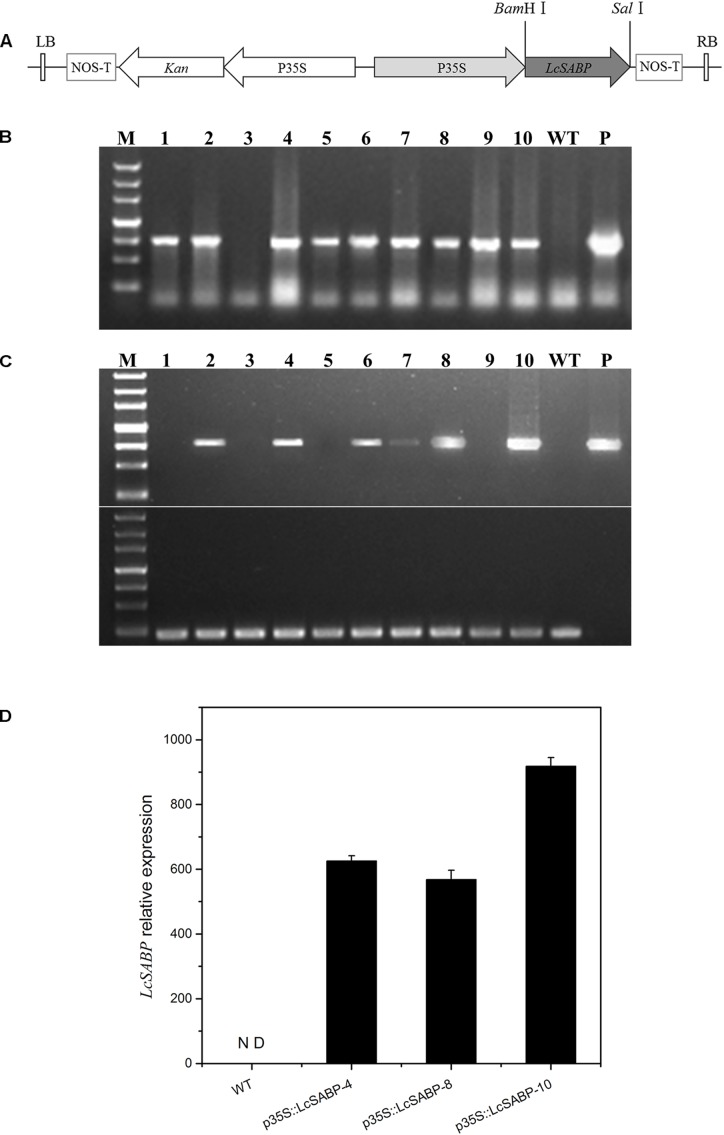
Molecular identifications of transgenic *LcSABP* tobacco lines via PCR. **(A)** The T-DNA region of the binary vector employed for *Agrobacterium tumefaciens*-mediated transformation. The Binary vector p35S::LcSABP containing *LcSABP* was driven by the CaMV35S promoter. *LcSABP*, salicylic acid binding protein gene from *L. chinense*; P35S, cauliflower mosaic virus-CaMV35S RNA promoter; NOS-T, 3′ terminator region of the nopaline synthase gene; Kan, kanamycin selection marker; LB, left border; RB, right border. **(B)** PCR results of primary transformants using specific primers for the *LcSABP* gene. It displays the PCR amplification of a 795 bp fragment of the *LcSABP* gene in transgenic lines. Lane 1, molecular marker; lanes 2–11, genomic DNA from putative transformants; lane 12, untransformed control; lane 13, p35S::LcSABP. **(C)** RT-PCR analysis of *LcSABP* expression in transgenic plants using specific primers for the *LcSABP* gene. Lane 1, molecular marker; lanes 2–11, cDNA from putative transformants; lane 12, untransformed control; lane 13, p35S::LcSABP. The *NtActin* gene served as the internal control. **(D)** qRT-PCR analysis of T2 transformants using quantified primers for p35S::LcSABP. WT, untransformed control. ^∗∗^Significantly different at the *P* < 0.01 level compared to WT.

To examine the roles of *LcSABP* in tobacco plants, a plant expression vector harboring p35S::LcSABP was constructed (Figure 3A) to express *LcSABP* under the control of constitutive CaMV35S promoter. The putative transgenic plants harboring p35S::LcSABP were selected on kanamycin, and were further confirmed using PCR with gene-specific primers (Figure 3B and Table 1). The 795 bp *LcSABP* cDNA was amplified from the transgenic plants (p35S::LcSABP) under normal conditions using RT-PCR (Figure 3C). Three independent transgenic lines (p35S::LcSABP-4, 8, and 10) with high levels of *LcSABP* expression were selected for further analysis. The qRT-PCR data displayed a 600–1000 fold up-regulation of *LcSABP* expression in the transgenic lines compared to that in the WT plants (Figure 3D).

### Phenotypic Changes and Drought Tolerance in Transgenic *N. tabacum*

**FIGURE 4:**
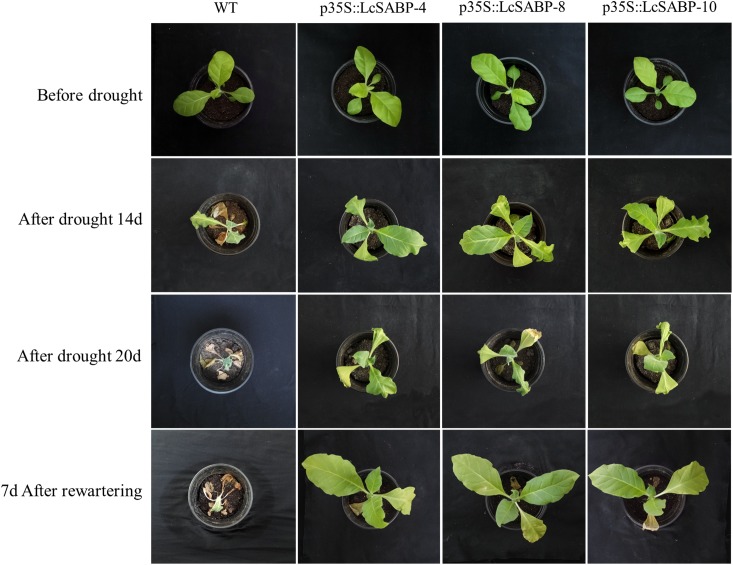
Morphological changes in WT and transgenic plants under drought stress. WT, wild type. The transgenic lines are indicated by the construct names followed by the line number: p35S::LcSABP-4 refers to p35S::LcSABP line 4, p35S::LcSABP-8 refers to p35S::LcSABP line 8 and p35S::LcSABP-10 refers to p35S::LcSABP line 10.

To investigate whether the *LcSABP* expression driven by CaMV35S promoter increases the drought stress tolerance of plants, WT and transgenic plants were subjected to drought stress for 20 days. Before exposure to drought stress, WT and transgenic plants were watered adequately and were observed to grow normally (Figure 4). On day 14 of drought stress treatment, the WT plants showed severe wilting. In comparison, the transgenic plants showed slight wilting. After 20 days of drought stress treatment, all the plants of WT and transgenic lines showed severe wilting. All the plants were re-watered on day 20 of drought stress treatment. After 7 days of re-watering, the transgenic plants recovered quickly, whereas the WT plants could not recover and eventually died (Figure 4).

### Overexpressing *LcSABP* Increases RWC, Proline, Chlorophyll Content and Decreases MDA Content in Transgenic *N. tabacum* Plants Exposed to Drought Stress

**FIGURE 5:**
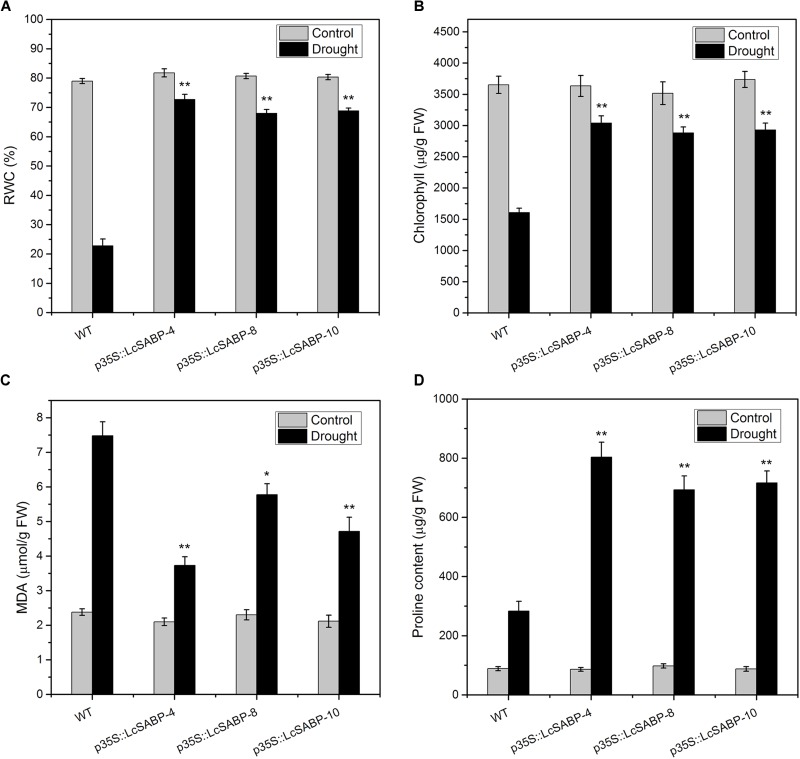
Effects of drought stress on relative water content (RWC) **(A)**, chlorophyll content **(B)**, and malondialdehyde (MDA) levels **(C)** proline content **(D)** in WT and transgenic tobacco plants after withholding watering for 14 days. WT, wild type. The transgenic lines were indicated by the construct names followed by the line number: p35S::LcSABP-4 refers to p35S::LcSABP line 4; p35S::LcSABP-8 refers to p35S::LcSABP line 8; p35S::LcSABP-10 refers to p35S::LcSABP line 10. Bars represent the mean ± SE of three independent experiments. ^∗∗^Significantly different at the *P* < 0.01 level compared to WT.

The RWC is considered to be a relevant tool for the measurement of drought tolerance, and provides a credible parameter for evaluation of the water status of plants (Flower and Ludlow, 2010). The RWC and chlorophyll content of tobacco plants on day 14 of drought stress treatment were determined. As shown in Figure 5A, the RWC was higher in all the *LcSABP* transgenic lines compared to that in the WT lines. The chlorophyll content, which is an important indicator of the photosynthetic efficiency of plants, was significantly higher in the transgenic lines than that in the WT lines, and the p35S::LcSABP-10 line showed the highest level (Figure 5B). The MDA content, which is an indicator of lipid peroxidation, was also measured in the leaves of transgenic and WT plants to detect the extent of damage in plants. On day 14 of drought stress treatment, significantly lower MDA levels were detected in the transgenic plants compared to that in the WT plants (Figure 5C). Proline is an important substance for plant osmotic adjustment, contributes to increase plant cell solute concentration, reduce osmotic potential and alleviate dehydration stress. Our data showed that the proline content in the transgenic lines was significantly higher than that in the WT lines (Figure 5D).

### Overexpression of *LcSABP* Increases Photosynthetic Capacity of Transgenic *N. tabacum* Plants Exposed to Drought Stress

**FIGURE 6:**
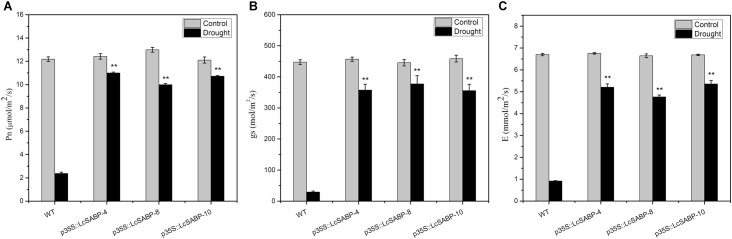
Effects of drought stress on leaf gas exchange parameters in WT and transgenic tobacco plants after withholding watering for 14 days. **(A)** Net photosynthesis rate (*P*_n_). **(B)** Stomatal conductance (*g*_s_). **(C)** Transpiration rate (*E*). WT, wild type. The transgenic lines were indicated by the construct names followed by the line number: p35S::LcSABP-4 refers to p35S::LcSABP line 4; p35S::LcSABP-8 refers to p35S::LcSABP line 8; p35S::LcSABP-10 refers to p35S::LcSABP line 10. Bars represent the mean ± SE of three independent experiments. ^∗∗^Significantly different at the *P* < 0.01 level compared to WT.

To evaluate the photosynthetic capacity of the WT and transgenic lines, the maximum net photosynthetic rate (*P*_n_), transpiration rate (*E*), and stomatal conductance (*g*_s_)-values of the plants were measured using a portable photosynthetic system (LI-6400XT) after 14 days of drought stress. The results showed that these indicators were almost indistinguishable the between WT and transgenic lines under adequate watering conditions. However, under drought stress, the *P*_n_, *E*, and *g*_s_ values decreased significantly in the WT lines, whereas those in the *LcSABP* transgenic lines showed only a slight decrease, indicating that the transgenic plants were less affected by drought stress. The largest difference between the transgenic and WT plants was observed after 14 days of drought stress (Figure 6).

### Overexpression of *LcSABP* Reduces ROS Accumulation in Transgenic *N. tabacum* Plants Exposed to Drought Stress

**FIGURE 7:**
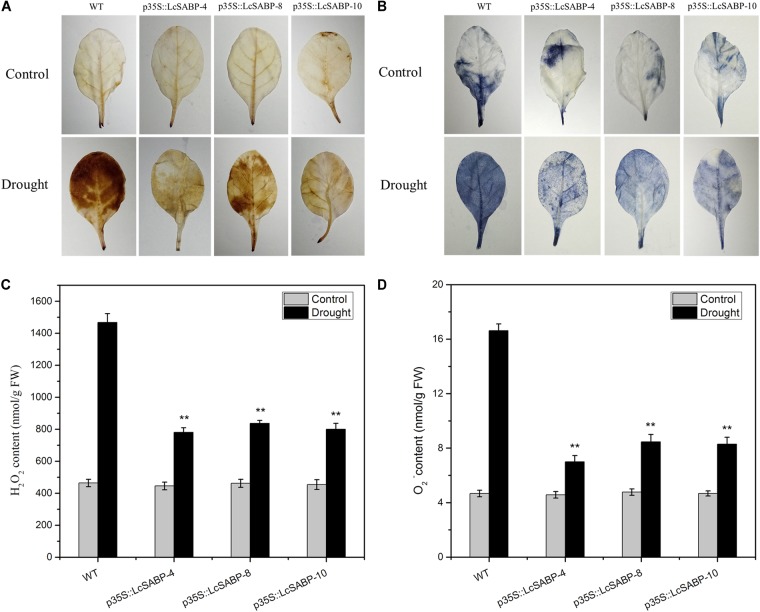
Changes in H_2_O_2_ **(A,C)** and O_2_^-^ **(B,D)** levels in WT and transgenic plants subjected to drought stress. Drought-stressed leaves were incubated in diaminobenzidine (DAB) or nitroblue tetrazolium (NBT) solution. Brown staining indicates H_2_O_2_ accumulation **(A)**. Blue staining indicates the location and levels of O_2_^-^**(B)**. H_2_O_2_ content **(C)** and O_2_^-^ content **(D)** were measured after drought treatment. The labels under the graphs correspond to the plant materials shown in **(A,B)**. Control, plants grown under normal conditions; drought, plants grown under drought stress treatment (14 days); WT, wild type. The transgenic lines were named by the construct names followed by the line number: p35S::LcSABP-4 refers to p35S::LcSABP line 4; p35S::LcSABP-8 refers to p35S::LcSABP line 8; p35S::LcSABP-10 refers to p35S::LcSABP line 10. Bars represent the mean ± SE of three independent experiments. ^∗∗^Significantly different at the *P* < 0.01 level compared to WT.

We further investigated whether the overexpression of *LcSABP* reduces ROS accumulation in plants exposed to drought stress. For this, H_2_O_2_ and O_2_^-^ accumulation in plants was detected using diaminobenzidine (DAB) and nitro blue tetrazolium (NBT) staining, respectively. Under conditions of adequate watering, few DAB staining spots were detected in the WT and transgenic plants. On day 14 of drought stress treatment, clear DAB staining was detected in the WT plants, whereas the staining much less in the transgenic plants (Figure 7A). These results indicated a lower level of H_2_O_2_ present in the transgenic lines compared to that in the WT lines. Similarly, the results of NBT staining revealed very less staining in both the WT and transgenic plants under conditions of adequate watering. The staining was distinct in the WT lines after 14 days of drought stress treatment, clear NBT staining was detected in the WT plants, whereas the staining was very less in the transgenic plants (Figure 7B), indicating that the O_2_^-^ levels in the transgenic plants were lower than in the WT plants. The interpretation from the results of staining was confirmed by quantitative analysis of H_2_O_2_ and O_2_^-^ levels, which indicated that the transgenic plants produced significantly lower levels of ROS than the WT plants (Figure 7C,D).

### Expression of *LcSABP* Affects the Activities of SOD, CAT, and APX

**FIGURE 8:**
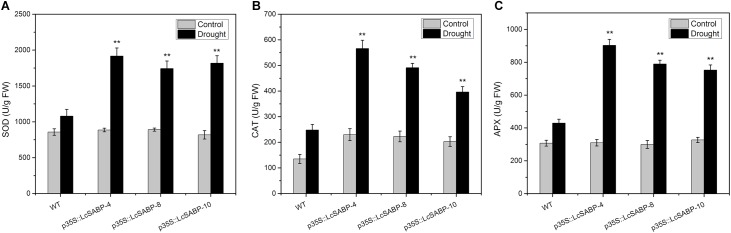
Superoxide dismutase (SOD) **(A)**, APX **(B)**, and CAT **(C)** enzyme activity in WT and transgenic tobacco plants after drought stress for 14 days. WT, wild type. The transgenic lines were indicated by the construct names followed by the line number: p35S::LcSABP-4 refers to p35S::LcSABP line 4; p35S::LcSABP-8 refers to p35S::LcSABP line 8; p35S::LcSABP-10 refers to p35S::LcSABP line 10. Bars represent the mean ± SE of three independent experiments. ^∗^Significantly different at the *P* < 0.05 level compared to WT. ^∗∗^Significantly different at the *P* < 0.01 level compared to WT.

The drought stress response of plants was further analyzed by determining the activities of SOD, CAT, and APX. These enzymes can eliminate the harmful accumulation of ROS under drought stress conditions. We observed that the trends of enzymatic activities were similar in all the plants exposed to drought stress (Figure 8A–C). Under normal conditions, the CAT activity in the *LcSABP* transgenic lines was slightly higher than that in the WT lines, whereas the SOD and APX activities were almost at the same in the WT and transgenic lines. Under drought stress conditions, the activities of all the antioxidant enzymes (SOD, CAT, and APX) in the *LcSABP* transgenic lines were higher than those in the WT lines, and no significant difference was detected among the *LcSABP* transgenic lines. These results indicated that enhanced drought tolerance in *LcSABP* transgenic lines was related to the increased activities of the antioxidant enzymes.

### *LcSABP* Regulates the Expression of Stress-Related Genes in Plants Exposed to Drought Stress

**FIGURE 9:**
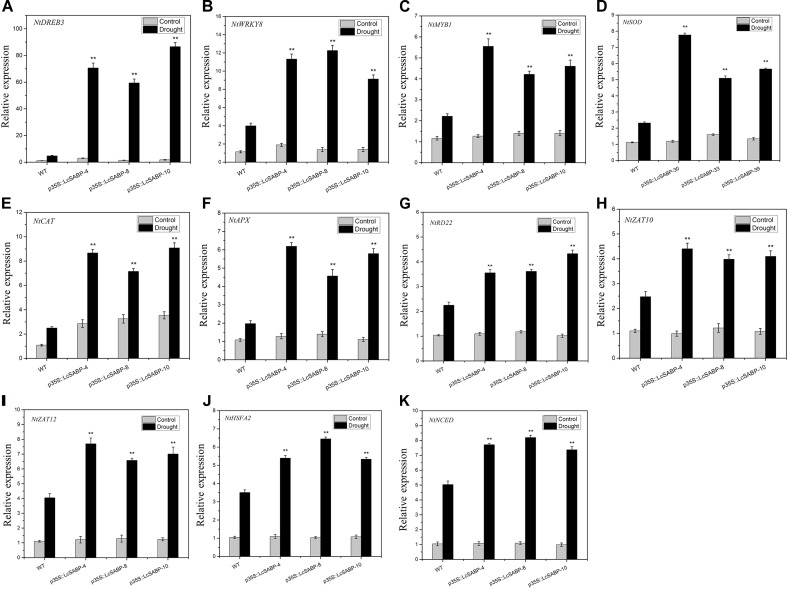
**(A–K)** Expression levels of abiotic stress-related genes and ROS-related genes in the WT and transgenic lines (p35S::LcSABP-4, p35S::LcSABP-8, and p35S::LcSABP-10) under drought stress. Expression levels were measured using cDNA synthesized from total RNA extracted from tobacco leaves, which suffered drought stress for 14 days. *NtActin* was used as the internal control. Bars represent the mean ± SE of three independent experiments. ^∗∗^Significantly different at the *P* < 0.01 level compared to WT.

To reveal the molecular mechanism of drought tolerance in transgenic *LcSABP* plants, qRT-PCR was carried out to assess the expression levels of stress-related genes in both the WT and transgenic plants before and after drought stress. These genes encoded key enzymes involved in ABA biosynthesis (NtNCED1), ROS detoxification (NtSOD, NtCAT, and NtAPX), as well as stress-responsive proteins (NtDREB3, NtWRKY8, NtMYB1, NtRD22, NtZAT10, NtZAT12, and NtHSFA2). The *NtActin* gene was used as an internal control. Under normal conditions, only the transcription levels of *NtCAT* were slightly higher in the transgenic plants compared to that in the WT plants (Figure 9E). Under drought stress conditions, the transcription levels of *NtDREB3, NtWRKY8, NtMYB1, NtSOD, NtCAT, NtAPX, NtRD22, NtZAT10, NtZAT12, NtHSFA2*, and *NtNCED1* were significantly increased in the transgenic plants compared to those in the WT plants (Figure 9A–K). Hence, the *LcSABP* transgenic plants had enhanced drought tolerance possibly as a result of the upregulation of ROS-related and stress-related genes.

### Extraction and Quantification of Endogenous SA and MeSA

**FIGURE 10:**
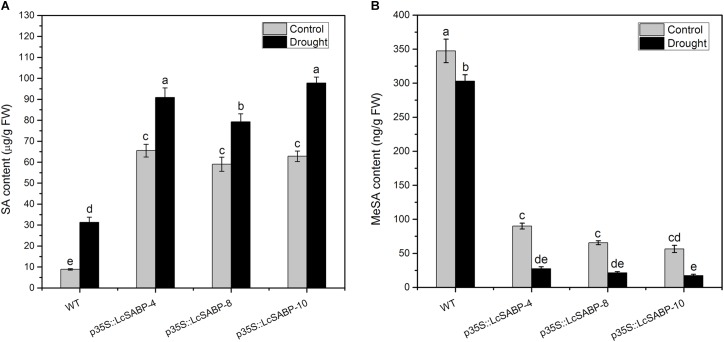
Salicylic acid biosynthesis capacity in WT and transgenic lines (p35S::LcSABP-4, p35S::LcSABP-8, and p35S::LcSABP-10). **(A)** Detection of SA content. **(B)** Detection of MeSA content. SA, salicylic acid; MeSA, methyl salicylate; FW, fresh weight. The leaves from tobacco plants were sampled for SA and MeSA analysis before and after 14-day water loss. WT, wild type. The transgenic lines were indicated by the construct names followed by the line number: p35S::LcSABP-4 refers to p35S::LcSABP line 4; p35S::LcSABP-8 refers to p35S::LcSABP line 8; p35S::LcSABP-10 refers to p35S::LcSABP line 10. Bars represent the mean ± SE of three independent experiments, columns labeled with distinct lowercase letters indicate statistically significant differences among treatments (*P* < 0.05).

Among the signaling molecules, SA has gradually attracted much attention because as a signal molecule it can endow the ability to resist biotic and abiotic stresses on plants by regulating the physiological and biochemical processes via its interaction with other substances in plants (Horvath et al., 2007; Szepesi et al., 2009; Asensi-Fabado et al., 2012). In this study, the accumulation of SA and MeSA was determined in the WT and transgenic plants under normal and drought conditions. The results showed that the overexpression of *LcSABP* induced more SA accumulation and lower MeSA content in the transgenic lines compared to that in the WT lines (Figure 10). The content of SA increased in all the transgenic and WT lines under drought conditions, but in the transgenic plants SA was significantly increased, whereas only a slight increase was observed in the WT plants (Figure 10A). In addition, after imposition of drought stress, the levels of MeSA were decreased in all the transgenic and WT lines, however, the decrease was significant only in the transgenic lines (Figure 10B). Hence, it is reasonable to conclude that the overexpression of *LcSABP* promoted more conversion of MeSA to SA in the transgenic plants. Therefore, quantification of SA and MeSA revealed that the overexpression of *LcSABP* induced enhanced accumulation of SA and less production of MeSA in the transgenic lines than in the WT lines. This suggests that enhanced drought tolerance of transgenic plants could be related to the accumulation of SA.

### Determination of ABA Content and Stomatal Aperture

**FIGURE 11:**
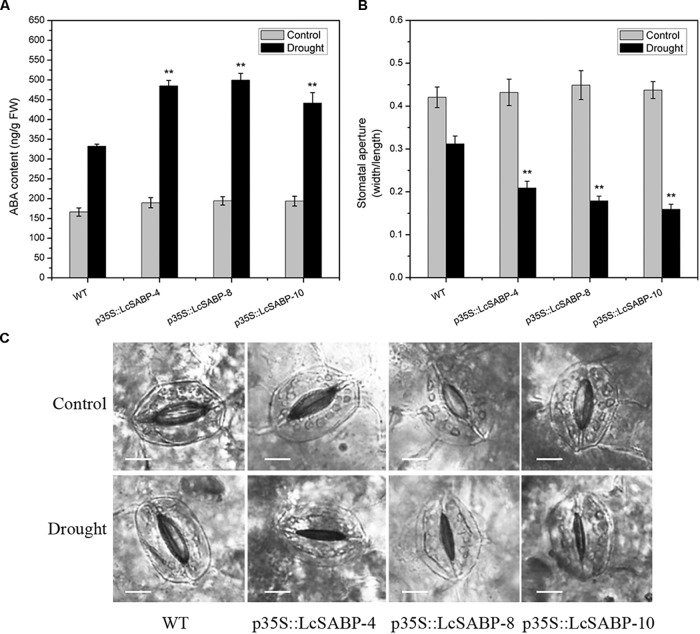
Changes in endogenous ABA content and stomatal aperture in WT and transgenic lines (p35S::LcSABP-4, p35S::LcSABP-8, and p35S::LcSABP-10). **(A)** Detection of ABA content. **(B)** Stomatal aperture after drought stress treatment. **(C)** Statistical analysis of the stomatal aperture. Scale bars = 10 μm. The leaves from tobacco plants were sampled for ABA content and stomatal aperture detection analysis before and after 14-day water loss. WT, wild type. The transgenic lines were indicated by the construct names followed by the line number: p35S::LcSABP-4 refers to p35S::LcSABP line 4; p35S::LcSABP-8 refers to p35S::LcSABP line 8; p35S::LcSABP-10 refers to p35S::LcSABP line 10. Bars represent the mean ± SE of three independent experiments, ^∗∗^Significantly different at the *P* < 0.01 level compared to WT.

The phytohormone ABA plays a critical role in regulating a range of plant physiological processes in response to various abiotic stresses (Huiru et al., 2014; Ijaz et al., 2017). Previous studies reported that drought stress caused significant accumulation of ABA and then this increased endogenous ABA content can induce stomatal closure (Hirayama and Shinozaki, 2007; Chu et al., 2015). In our study, the endogenous ABA content was measured in WT and transgenic plants. The results showed that, when exposed to drought stress, the ABA level was all increased in transgenic and WT lines, and accumulation of ABA in transgenic lines was significantly higher than in WT lines when exposed to drought treatment (Figure 11A). Then, the stomatal aperture was measured in WT and transgenic lines under control and drought treatments. Under normal conditions, no significant difference of stomatal apertures in WT and transgenic plants was observed. However, transgenic plants showed significant reduction of stomatal apertures than WT plants after drought treatment (Figure 11B,C).

## Discussion

Water deficit is a major abiotic stress factor that limits crop yields by affecting cell division, root growth, stem elongation, leaf expansion, and inhibition of the absorption of water and nutrients, ultimately affecting the income of farmers in arid and semi-arid regions (Bhatnagar-Mathur et al., 2007; Zhu et al., 2016). A number of signaling mechanisms have developed in plants over the long period of evolution to adapt to and resist various stresses. Among the signaling molecules, SA has gradually become a focus of research because it can endow plants with the ability to resist biotic and abiotic stresses by regulating several physiological and biochemical processes (Jayakannan et al., 2015). Previous research has mainly focused on the physiological response of plants to exogenous SA application, and functional analysis of the roles of endogenous SA in regulating the response of plants to drought stress has not been undertaken (Fayez and Bazaid, 2014). The involvement of SABP2 in SA production in plants, such as *Arabidopsis* and tobacco, under biotic stresses has been well documented, but there are few reports in *L. chinense* (Vlot et al., 2008; Song et al., 2009). In this study, a *SABP2*-like gene, *LcSABP*, was cloned from *L. chinense*. The conserved domain of LcSABP was analyzed and it was observed that it belonged to the alpha/beta hydrolase family with MeSA esterase activity. At the amino acid level, LcSABP showed high sequence identity with SABP2 orthologs from other plants. The phylogenetic analysis revealed that LcSABP had a closer relationship with the SABP2 from *N. tabacum* than with the orthologs from other plant species (Figure 1B). As evident from the qRT-PCR results, *LcSABP* was more abundantly expressed in the roots (Figure 2A). The characteristic organ-specific expression of *LcSABP* might be related to the differences in the species and time of sampling. The expression level of *LcSABP* under drought stress treatment in Figure 2B indicated that *LcSABP* expression might be involved in the regulation of drought stress signals. Our results showed that the overexpression of *LcSABP* contributes to a substantial improvement in the ability of tobacco plants to survive under drought stress conditions (Figure 4), suggesting that *LcSABP* plays an important role in drought tolerance of plants.

The manipulation of SA biosynthesis is considered to be a biotechnological tool for enhancing stress tolerance of crops. The overproduction of SA by enhancing the activities of enzymes of the SA biosynthetic pathway was shown to contribute to the tolerance of plants to environmental stress (Slaymaker et al., 2002; Lin et al., 2013). Biological analysis revealed that loss of SA accumulation resulted in a decrease in pathogen resistance in *A. thaliana* (Koo et al., 2007). The accumulation of SA might trigger stress response in plants (Xia et al., 2015). The analysis of tobacco plants initially suggested that SAR was activated in the following sequence: SA accumulated in the pathogen-inoculated leaves and was converted to MeSA by NtSAMT1, MeSA then traveled to vitreous via the phloem, in the systemic leaves, MeSA was converted to SA by the methyl esterase activity of NtSABP2, and the increased accumulation of SA triggered the systemic defense (Strawn et al., 2007; Dempsey et al., 2011). It could be worth noting that SABP2 catalyzed the conversion from MeSA to SA. Furthermore, there have been increasing evidences proving that endogenous SA is employed to improve the growth and development of plants under abiotic stress (Slaymaker et al., 2002; Miura and Tada, 2014). Therefore, determination of the endogenous content of SA and MeSA was carried out to investigate the roles of *LcSABP* in the drought tolerance of plants. It was observed that the transgenic lines had significantly higher SA levels and much lower MeSA levels than the WT lines (Figure 10). Although the level of SA increased in all the transgenic and WT lines under drought stress conditions, the increase was significant only in the transgenic lines (Figure 10A), and the slight increase observed in the WT plants was not sufficient for drought tolerance of plants (Figure 4). After drought treatment, the level of MeSA decreased in all the transgenic and WT lines, but the decrease was significant only in the transgenic plants (Figure 10B). These results indicated that LcSABP probably catalyzed more conversion of MeSA to SA, and the increased accumulation of SA triggered significant drought tolerance in the transgenic plants (Figure 4). Thus, the overexpression of *LcSABP* significantly increased the endogenous SA content in tobacco plants. In addition, the slight increase in SA accumulation in WT plants under drought stress conditions indicated that SA might be induced by drought stress, as has been suggested in previous studies (Marini, 2008; Farooq et al., 2012). The higher levels of SA in the *LcSABP*-overexpressing plants further improved the drought tolerance of transgenic tobacco plants as shown in Figure 4.

Under normal physiological conditions, the production of ROS during plant metabolism is an inevitable result of electron transport systems, and adverse environmental conditions (such as drought) can stimulate the production of ROS (such as H_2_O_2_ and O_2_^-^) in plants, leading to photo-oxidative stress, and can further lead to the degradation of photosynthetic pigments and decreased photosynthetic capacity of plants (Foyer et al., 1994). The ROS homeostasis is closely associated with oxidative stress. The ROS-induced oxidative stress is damaging to plants and decreasing this stress would enhance their growth. Drought stress promotes the overproduction of ROS in plants. H_2_O_2_ and O_2_^-^ are among the major ROS generated in plants under drought stress, they are highly toxic and cause damage to proteins, lipids, and other biomacromolecules (Wahid, 2009). Therefore, we determined the levels of ROS, which reflect the degree of drought stress damage to plant cells. The contents of H_2_O_2_ and O_2_^-^ were detected to be lower in the transgenic plants compared to that in the WT plants, indicating that the transgenic plants which contained higher SA content could scavenge more ROS compared to the WT plants exposed to drought stress (Figure 7). Consistent with our results, both endogenous and exogenous SA was evidenced to play roles in antioxidant metabolism and have a tight control over cellular ROS (Kang et al., 2014; Khan et al., 2014). In a previous study, it was proved that SA alleviates cadmium-induced inhibition of photosynthesis through upregulation of antioxidant defense system in two melon cultivars (*Cucumis melo* L.). These findings indicated that SA might play a positive role in alleviating photo-oxidative damage and in protecting photosynthetic capacity of plants. In addition, the photosynthetic capacity can also be compromised by damage to the photosynthetic apparatus, which involves sensitive pigments, photosystems, and chloroplast ultrastructure (Gong et al., 2014; Wei et al., 2016). Previous studies have shown that lower RWC inhibited and seriously affected the photosynthesis in plants (Flexas and Medrano, 2002). We observed that the increased RWC in *LcSABP*-overexpressing plants (Figure 5A) was associated with enhanced photosynthesis (Figure 6). Furthermore, recent evidences also indicated that SA could protect the photosynthesis process (Khan et al., 2014), and was an important regulator of photosynthesis, photosystem II (PSII), and photosynthetic pigment under heavy metal stress (Khan et al., 2015; Zhang et al., 2015). Another indicator of drought tolerance is the accumulation of photosynthetic pigments, such as chlorophyll. Chlorophyll content is considered to be a biochemical indicator of drought tolerance in different crops, because it is vital for carbon fixation and is involved in capturing the solar radiation that drives photosynthesis. Drought stress was reported to severely decrease the contents of chlorophyll a and chlorophyll b in marigold (Asrar and Elhindi, 2011; Farooq et al., 2012). In the present study, the p35S::LcSABP transgenic plants showed higher chlorophyll content (Figure 5B), which might be partly responsible for the higher *P*_n_ in transgenic lines under drought conditions (Figure 6A) (Begcy et al., 2011; Gong et al., 2014). Further, we determined the levels of proline, which is an important compatible solute that accumulates in plant tissues under abiotic stress conditions. High levels of proline can maintain low water potential in plants, promoting water uptake from the environment (Pál et al., 2010). In our study, the transgenic plants showed higher accumulation of proline than the WT plants exposed to drought stress (Figure 5D), indicating osmoprotection and restoration of tissue water, which increased the RWC in transgenic plants and is in agreement with the results presented in Figure 5A. Drought tolerance is also associated with stomatal control. In the transgenic lines, higher stomatal conductance and transpiration rates stimulated photosynthesis during dry periods (Figure 6B,C). To decipher the other probable mechanisms responsible for improved drought tolerance of *LcSABP*-overexpressing transgenic plants, we further explored the physiological differences between the transgenic and WT plants under drought stress. The improvement in the growth of barley plants on saline soils as a result of the ability of SA to decrease the contents of MDA and ROS (such as H_2_O_2_) has been reported (Fayez and Bazaid, 2014). The MDA content is often used as an indicator of oxidative damage caused by enhanced generation of ROS (Miller et al., 2010; Fayez and Bazaid, 2014). In the present study, the WT lines were observed to have higher MDA content than the transgenic lines exposed to drought stress, indicating that the WT plants suffered more extensive oxidative damage compared to the transgenic plants under drought stress (Figure 5C). All the results described above indicated that transgenic plants with higher SA contents showed higher photosynthetic capacity and lower photo-oxidative damage, which was attributed to the increased RWC and chlorophyll content, decreased MDA, H_2_O_2_, and O_2_^-^ content by *LcSABP* (Figure 5, 7). Therefore, we surmise that SA plays importantly positive role in the signaling of plants’ response to photo-oxidative stress. This also provides evidence for the probable mechanisms through which *LcSABP* ameliorates drought stress-induced damage.

Abscisic acid acts as an important regulator and plays a key role in abiotic stress signaling (Xiao-Yun et al., 2013). Previous study reported that SA treatment increased the ABA content in the leaves of barley, which might have contributed to the development of the antistress reactions to drought stress of plants induced by SA (Bandurska and Ski, 2005). The increased salt tolerance in *Solanum lycopersicum* L. was related to accumulation of ABA stimulating by SA treatment (Szepesi et al., 2009). It was also found that SA-induced increase in ABA content of wheat seedlings under salinity, which might contributed to a preadaptation of plants to salt stress (Shakirova et al., 2003). Therefore, the content of ABA was measured in the present study. Consistent with previous studies, we found that the transgenic plants with increased SA content showed a significant increase in the accumulation of ABA compared to WT plants under drought stress. Our results indicated that the increased endogenous SA could stimulate higher accumulation of ABA in the transgenic plants compared to that in the WT plants and contributed to plants drought resistance (Figure 11A). However, antagonistic effects between ABA and SA were also reported before (Michiko et al., 2008). Thus, these findings indicated that the interaction of SA with other hormones like ABA in plants under abiotic stress is a complicated dynamic equilibrium process which needs further research. The above-mentioned data provided evidence to support the fact that endogenous ABA is a hormonal intermediate in the SA-induced protection of plants under abiotic stress. The rapid accumulation of ABA can lead to stomatal closure and the inhibition of stomatal opening, which increases the capacity for osmotic regulation and reduces water loss by transpiration in plants (Wei Q. et al., 2017). Similar results were found in our study, the transgenic lines showed enhanced stomatal closure compared to the WT line under drought stress (Figure 11B,C), and the enhanced stomatal closure contributes to the decreased water loss and enhanced drought tolerance capacity, consistent with our RWC data mentioned above (Figure 5A). Therefore, in the present study, the increased ABA content and less stomatal aperture suggested that *LcSABP* has a function in regulating stomatal movement and, thus, might improve drought resistance at least partially by ABA signaling.

Efficient antioxidant mechanisms, including those requiring enzymatic antioxidants, evolved in plants to detoxify the ROS and to minimize the oxidative damage. Enzymatic antioxidants mainly include SOD, CAT, and APX (Habib et al., 2016; Nath et al., 2016). It is established that SOD is the first line of defense against oxygen free radicals, and it catalyzes the dismutation of H_2_O_2_, the products of which are then scavenged by coordinated action of CAT and APX (Shafi et al., 2015; You and Chan, 2015; Wang et al., 2018). The antioxidative systems in plants keep ROS at relatively low non-toxic levels under normal conditions (Mittler, 2016). Previous studies have shown that SA-pretreatment enhances photosynthesis and growth in *Vigna radiata* under salt stress through the enhancement of the activities of antioxidant enzymes (such as SOD, CAT, and APX) (Mutlu et al., 2013; Khan et al., 2014, 2015). Our data showed that the SOD, CAT, and APX activities in the transgenic lines were similar as those in the WT lines in the absence of drought stress (Figure 8A–C). To minimize the detrimental effects of oxidative damage, plants enhance the production of antioxidants to normalize their metabolic activities under drought-induced oxidative stress (Farooq et al., 2012). We observed higher activities of SOD, CAT, and APX in the transgenic lines compared to the corresponding activities in the WT lines. These data were consistent with the results of previous studies (Radhakrishnan and Lee, 2013; Kamiab et al., 2014; Duan et al., 2017; Wei Q. et al., 2017). Interestingly, under normal conditions, only the CAT activity was slightly higher in the transgenic lines (Figure 8B), whereas the SOD and APX activities showed no significant difference between the WT and transgenic plants (Figure 8A,C). The data indicated that the overexpression of *LcSABP* slightly enhanced the CAT activity in the transgenic plants. Our results demonstrate that *LcSABP* expression under the control of CAMV35S promoter enhanced the drought tolerance of tobacco plants by stimulating the antioxidant defense system.

The exogenous application of SA (3 mM) was reported to increase the tolerance of rice to cadmium stress, and it also enhanced the expression of *OsWRKY45* in rice (Chao et al., 2010). The WRKY TFs genes play significant roles in the response of plants to abiotic stress (Chen et al., 2012; Shi et al., 2014). Previous studies have revealed that the overexpression of wheat *WRKY* genes, *TaWRKY2* and *TaWRKY19*, improved the salt and drought tolerance of transgenic *Arabidopsis* plants (Niu et al., 2012). To further investigate the mechanism of enhancement of drought tolerance by *LcSABP* at the molecular level, the expression levels of genes encoding stress-responsive TFs and antioxidant defense-related proteins were examined before and after the imposition of drought stress. TFs play significant roles in the response of plants to abiotic stress (Qin et al., 2013; Wu et al., 2014; Han et al., 2018). The dehydration responsive element binding proteins (DREB) belong to the plant-specific APETALA2(AP2)/ethylene-responsive element-binding protein (EREBP) family of TFs, and DREB TFs are proved to contribute to abiotic stress response in different plant species (Wei T. et al., 2017). In the present study, *NtDREB3* was found to be up-regulated in the transgenic plants under drought stress (Figure 9A), which implied that *LcSABP* activated the DREB-mediated drought stress defense response. The transcriptional levels of *NtWRKY8* were up-regulated in the *LcSABP* transgenic lines compared to those in the WT lines (Figure 9B), implying that WRKY family members may be induced by *LcSABP* in the transgenic lines under drought stress. The huge MYB superfamily in plants is involved in the mediation of stress response in plants (Dubos et al., 2010; Ambawat et al., 2013). Our results showed that the levels of *NtMYB1* were significantly higher in the *LcSABP*-overexpressing plants than that in the WT plants under drought stress (Figure 9C). These results suggested that increased synthesis of NtMYB1 in transgenic plants might be useful for drought tolerance. *NtSOD, NtCAT*, and *NtAPX* encode the antioxidant enzymes SOD, CAT, and APX, respectively, and these enzymes are involved in ROS detoxification. Under normal conditions, the transcript levels of *NtCAT* were slightly higher in the transgenic lines than that in the WT lines, which was consistent with the changes in the CAT activity (Figure 9E). The transcription of *NtSOD, NtCAT*, and *NtAPX* was up-regulated in the transgenic plants compared to that in the WT plants under drought stress (Figure 9D–F). These findings were consistent with the results of antioxidant enzyme activities described above (Figure 8), implying that the overexpression of *LcSABP* in plants might regulate the activities of antioxidant enzymes by transcriptionally modulating the expression of genes involved in ROS scavenging. In our study, the increase of endogenous SA in transgenic plants induced more accumulation of ABA after drought stress (Figure 11A). It is reported that *NCED* and *RD22* genes accumulated in plants that had been exposed to ABA or drought stress (Hiroshi et al., 2003; Ding et al., 2009; Chen et al., 2015). The expression levels of *NtNCED* and *NtRD22* were measured in our study. The results showed that *NtRD22* was up-regulated in transgenic lines compared with WT lines exposed to drought stress (Figure 9G). In addition to *NtRD22*, drought stress also leaded to the up-regulation of *NtNCED* involved in ABA biosynthesis in transgenic plants compared with WT plants (Figure 9K), which is consistent with the results of its product ABA accumulation in plants (Figure 11A). This indicated that the enhanced drought tolerance conferred by *LcSABP* overexpression might be at least partially attributed to the increased ABA biosynthesis and signaling. In *Arabidopsis*, the zinc-finger TF, ZAT10/12, could specifically activate the ROS-related antioxidant defense genes, e.g., cytosolic *APX1* and *FeSOD1*, playing a key role in abiotic stress tolerance (Miller et al., 2008; Makita and Gonda, 2012; Mehterov et al., 2012). *HSFA2* is an inducer of heat shock gene, which confers tolerance to several abiotic stresses and is shown to function upstream to the ROS scavenging systems in *Arabidopsis* (Li et al., 2005; Banti et al., 2010; Mehterov et al., 2012). In a previous study, it was reported that *AtHsfA2* modulated the expression of stress responsive genes, such as *APX1*, a central component of the reactive oxygen gene network, enhanced tolerance to heat and oxidative stress in *Arabidopsis* (Li et al., 2005). In the present study, the transcriptional levels of *NtZAT10*/*12* and *NtHSFA2* were up-regulated in the *LcSABP* transgenic lines compared to those in the WT lines (Figure 9H–J). These findings implied that the increase in endogenous SA in transgenic plants might stimulate *NtZAT10*/*12* and *NtHSFA2*, which function in ROS scavenging systems for counteracting ROS burst to enhance drought tolerance in plants. The qRT-PCR results demonstrated that the molecular mechanism of enhanced drought tolerance in transgenic plants not only relied on the induction of ROS-related genes but also of TFs genes involved in signal transduction in the stress response of plants.

## Conclusion

The overexpression of *LcSABP* under the control of CAMV35S promoter significantly enhanced the drought tolerance of tobacco plants. Furthermore, the overexpression of *LcSABP* significantly elevated the content of SA and decreased the content of MeSA in the transgenic plants. In addition, the *LcSABP*-overexpressing plants showed higher photosynthesis; they had higher RWC, chlorophyll and ABA content, lower MDA, H_2_O_2_, and O_2_^-^ contents, and higher activities of antioxidant enzymes. The expression of ROS-related and stress responsive TFs genes was also higher in the transgenic plants under drought stress. It is concluded that *LcSABP*, an orthologous gene of SA binding protein 2, isolated from *L. chinense* enhances drought stress tolerance in transgenic tobacco plants. *LcSABP* might be involved in the tolerance to drought stress via an SA-dependent pathway, which could play an important role in the antioxidant mechanism at the transcriptional and translational levels. In addition, ABA quantification, combined with expression levels of *NtNCED1* and *NtRD22*, demonstrated that overexpression of *LcSABP* might enhance plants drought resistance at least partially by ABA signaling. However, the molecular basis of the enhanced drought stress tolerance and the related metabolic regulatory pathways remain poorly understood. More in-depth research, including transcriptomic and metabolomic studies, should be carried out to explore more target genes regulated by *LcSABP* and to decipher other hormone signaling network in plants that are modulated by endogenous SA. These studies would help in elucidating the function of *LcSABP*-mediated plant hormone homeostasis in the drought tolerance response of plants.

## Author Contributions

QL, GW, and CG designed the experiments, analyzed the data, and wrote the manuscript. QL performed the main experiments in this study. DY, YZ, and TA helped in the stress experiments. YW, JJ, and CJ contributed to data analysis and discussion. All authors read and approved the final manuscript.

## Conflict of Interest Statement

The authors declare that the research was conducted in the absence of any commercial or financial relationships that could be construed as a potential conflict of interest.

## Abbreviations

ABA: abscisic acid
APX: peroxidase
CAT: catalase
MDA: malondialdehyde
MeSA: methyl salicylate
ROS: reactive oxygen species
RWC: relative water content
SA: salicylic acid
SABP: salicylic acid binding protein
SOD: superoxide dismutase
TFs: transcription factors
WT: wild type

## References

[B1] Ambawat S. Sharma P. Yadav N. R. Yadav R. C. (2013). MYB transcription factor genes as regulators for plant responses: an overview. *Physiol. Mol. Biol. Plants* 19 307–321. 10.1007/s12298-013-0179-1 24431500 PMC3715649

[B2] An X. Liao Y. Zhang J. Dai L. Zhang N. Wang B. (2015). Overexpression of rice NAC gene *SNAC1* in ramie improves drought and salt tolerance. *Plant Growth Regul.* 76 211–223. 10.1007/s10725-014-9991-z

[B3] Asensi-Fabado M. A. Cela J. Muller M. Arrom L. Chang C. Munne-Bosch S. (2012). Enhanced oxidative stress in the ethylene-insensitive (ein3-1) mutant of *Arabidopsis thaliana* exposed to salt stress. *J. Plant Physiol.* 169 360–368. 10.1016/j.jplph.2011.11.007 22209220

[B4] Asensi-Fabado M. A. Munné-Bosch S. (2011). The aba3-1 mutant of *Arabidopsis thaliana* withstands moderate doses of salt stress by modulating leaf growth and salicylic acid levels. *J. Plant Growth Regul.* 30 456–466. 10.1007/s00344-011-9208-x

[B5] Ashraf M. Akram N. A. Arteca R. N. Foolad M. R. (2010). The physiological, biochemical and molecular roles of brassinosteroids and salicylic acid in plant processes and salt tolerance. *Crit. Rev. Plant Sci.* 29 162–190. 10.1080/07352689.2010.483580

[B6] Asrar A. W. Elhindi K. M. (2011). Alleviation of drought stress of marigold (*Tagetes erecta*) plants by using arbuscular mycorrhizal fungi. *Saudi J. Biol. Sci.* 18 93–98. 10.1016/j.sjbs.2010.06.007 23961109 PMC3730742

[B7] Bandurska H. Ski A. S. (2005). The effect of salicylic acid on barley response to water deficit. *Acta Physiol. Plant* 27 379–386. 10.1007/s11738-005-0015-5

[B8] Banti V. Mafessoni F. Loreti E. Alpi A. Perata P. (2010). The heat-inducible transcription factor HsfA2 enhances anoxia tolerance in *Arabidopsis*. *Plant Physiol.* 152 1471–1483. 10.1104/pp.109.149815 20089772 PMC2832282

[B9] Begcy K. Mariano E. D. Mattiello L. Nunes A. V. Mazzafera P. Maia I. G. (2011). An *Arabidopsis* mitochondrial uncoupling protein confers tolerance to drought and salt stress in transgenic tobacco plants. *PLoS One* 6:e23776. 10.1371/journal.pone.0023776 21912606 PMC3166057

[B10] Bhatnagar-Mathur P. Devi M. J. Reddy D. S. Lavanya M. Vadez V. Serraj R. (2007). Stress-inducible expression of *AtDREB1A* in transgenic peanut (*Arachis hypogaea* L.) increases transpiration efficiency under water-limiting conditions. *Plant Cell Rep.* 26 2071–2082. 10.1007/s00299-007-0406-8 17653723

[B11] Chao Y. Y. Chen C. Y. Huang W. D. Chinghuei K. (2010). Salicylic acid-mediated hydrogen peroxide accumulation and protection against Cd toxicity in rice leaves. *Plant Soil* 329 327–337. 10.1007/s11104-009-0161-4

[B12] Chen L. Song Y. Li S. Zhang L. Zou C. Yu D. (2012). The role of WRKY transcription factors in plant abiotic stresses. *Biochim. Biophys. Acta* 1819 120–128. 10.1016/j.bbagrm.2011.09.002 21964328

[B13] Chen T. Li W. Hu X. Guo J. Liu A. Zhang B. (2015). A cotton MYB transcription factor, GbMYB5, is positively involved in plant adaptive response to drought stress. *Plant Cell Physiol.* 56 917–929. 10.1093/pcp/pcv019 25657343

[B14] Chen Z. Klessig D. F. (1991). Identification of a soluble salicylic acid-binding protein that may function in signal transduction in the plant disease-resistance response. *Proc. Natl. Acad. Sci. U.S.A.* 88 8179–8183. 10.1073/pnas.88.18.8179 11607212 PMC52470

[B15] Chu X. Wang C. Chen X. Lu W. Li H. Wang X. (2015). The cotton WRKY gene *GhWRKY41* positively regulates salt and drought stress tolerance in transgenic *Nicotiana benthamiana*. *PLoS One* 10:e0143022. 10.1371/journal.pone.0143022 26562293 PMC4643055

[B16] Cramer G. R. Urano K. Delrot S. Pezzotti M. Shinozaki K. (2011). Effects of abiotic stress on plants: a systems biology perspective. *BMC Plant Biol.* 11:163. 10.1186/1471-2229-11-163 22094046 PMC3252258

[B17] Dempsey D. M. A. Vlot A. C. Wildermuth M. C. Klessig D. F. (2011). Salicylic acid biosynthesis and metabolism. *Arabidopsis Book* 9:e0156. 10.1199/tab.0156 22303280 PMC3268552

[B18] Ding Z. Li S. An X. Liu X. Qin H. Wang D. (2009). Transgenic expression of *MYB15* confers enhanced sensitivity to abscisic acid and improved drought tolerance in *Arabidopsis thaliana*. *J. Genet. Genomics* 36 17–29. 10.1016/S1673-8527(09)60003-5 19161942

[B19] Du H. Klessig D. F. (1997). Identification of a soluble, high-affinity salicylic acid-binding protein in tobacco. *Plant Physiol.* 113 1319–1327. 10.1104/pp.113.4.1319 12223676 PMC158255

[B20] Duan F. Ding J. Lee D. Lu X. Feng Y. Song W. (2017). Overexpression of *SoCYP85A1*, a spinach cytochrome p450 gene in transgenic tobacco enhances root development and drought stress tolerance. *Front. Plant Sci.* 8:1909. 10.3389/fpls.2017.01909 29209339 PMC5701648

[B21] Dubos C. Stracke R. Grotewold E. Weisshaar B. Martin C. Lepiniec L. (2010). MYB transcription factors in *Arabidopsis*. *Trends Plant Sci.* 15 573–581. 10.1016/j.tplants.2010.06.005 20674465

[B22] Engelberth J. Schmelz E. A. Alborn H. T. Cardoza Y. J. Huang J. Tumlinson J. H. (2003). Simultaneous quantification of jasmonic acid and salicylic acid in plants by vapor-phase extraction and gas chromatography-chemical ionization-mass spectrometry. *Anal. Biochem.* 312 242–250. 10.1016/S0003-2697(02)00466-9 12531212

[B23] Farooq M. Hussain M. Wahid A. Siddique K. H. M. (2012). “Drought stress in plants: an overview,” in *Plant Responses to Drought Stress: From Morphological to Molecular Features*, ed. Aroca R. (Berlin: Springer), 1–33.

[B24] Fayez K. A. Bazaid S. A. (2014). Improving drought and salinity tolerance in barley by application of salicylic acid and potassium nitrate. *J. Saudi Soc. Agric. Sci.* 13 45–55. 10.1016/j.jssas.2013.01.001

[B25] Flexas J. Medrano H. (2002). Energy dissipation in C3 plants under drought. *Funct. Plant Biol.* 29 1209–1215. 10.1071/FP02015 32689573

[B26] Flower D. J. Ludlow M. M. (2010). Contribution of osmotic adjustment to the dehydration tolerance of water-stressed pigeon pea (*Cajanus cajan* (L.) millsp.) leaves. *Plant Cell Environ.* 9 33–40. 10.1111/1365-3040.ep11589349

[B27] Forouhar F. Yang Y. Kumar D. Chen Y. Fridman E. Park S. W. (2005). Structural and biochemical studies identify tobacco SABP2 as a methyl salicylate esterase and implicate it in plant innate immunity. *Proc. Natl. Acad. Sci. U.S.A.* 102 1773–1778. 10.1073/pnas.0409227102 15668381 PMC547883

[B28] Foyer C. H. Lelandais M. Kunert K. J. (1994). Photooxidative stress in plants. *Physiol. Plant.* 92 696–717. 10.1111/j.1399-3054.1994.tb03042.x

[B29] Fritig B. Legrand M. (1993). *Mechanisms of Plant Defense Responses.* Dordrecht: Springer 10.1007/978-94-011-1737-1

[B30] Ghani A. K. I. (2015). Amelioration of lead toxicity in *Pisum sativum* (L.) by foliar application of salicylic acid. *J. Environ. Anal. Toxicol.* 5:292 10.4172/2161-0525.1000292

[B31] Gong B. Li X. VandenLangenberg K. M. Wen D. Sun S. Wei M. (2014). Overexpression of S-adenosyl-L-methionine synthetase increased tomato tolerance to alkali stress through polyamine metabolism. *Plant Biotechnol. J.* 12 694–708. 10.1111/pbi.12173 24605920

[B32] Habib S. H. Kausar H. Saud H. M. (2016). Plant growth-promoting rhizobacteria enhance salinity stress tolerance in okra through ROS-Scavenging enzymes. *Biomed Res. Int.* 2016:6284547. 10.1155/2016/6284547 26951880 PMC4756578

[B33] Han D. Zhang Z. Ding H. Chai L. Liu W. Li H. (2018). Isolation and characterization of *MbWRKY2* gene involved in enhanced drought tolerance in transgenic tobacco. *J. Plant Interact.* 13 163–172. 10.1080/17429145.2018.1447698

[B34] Hirayama T. Shinozaki K. (2007). Perception and transduction of abscisic acid signals: keys to the function of the versatile plant hormone ABA. *Trends Plant Sci.* 12 343–351. 10.1016/j.tplants.2007.06.013 17629540

[B35] Hiroshi A. Takeshi U. Takuya I. Motoaki S. Kazuo S. Kazuko Y. S. (2003). *Arabidopsis* AtMYC2 (bHLH) and AtMYB2 (MYB) function as transcriptional activators in abscisic acid signaling. *Plant Cell* 15 63–78. 10.1105/tpc.006130.salt 12509522 PMC143451

[B36] Horváth E. Szalai G. Janda T. (2007). Induction of abiotic stress tolerance by salicylic acid signaling. *J. Plant Growth Regul.* 26 290–300. 10.1007/s00344-007-9017-4

[B37] Horvath E. Szalai M. P. Paldi E. Janda T. (2007). Exogenous 4-hydroxybenzoic acid and salicylic acid modulate the effect of short-term drought and freezing stress on wheat plants. *Biol. Plant.* 51 480–487. 10.1007/s10535-007-0101-1

[B38] Huiru Y. Haihong J. Xiaobo C. Lili H. Hailong A. Xingqi G. (2014). The cotton WRKY transcription factor GhWRKY17 functions in drought and salt stress in transgenic *Nicotiana benthamiana* through ABA signaling and the modulation of reactive oxygen species production. *Plant Cell Physiol.* 55 2060–2076. 10.1093/pcp/pcu133 25261532

[B39] Ijaz R. Ejaz J. Gao S. Liu T. Imtiaz M. Ye Z. (2017). Overexpression of annexin gene *AnnSp2*, enhances drought and salt tolerance through modulation of ABA synthesis and scavenging ROS in tomato. *Sci. Rep.* 7:12087. 10.1038/s41598-017-11168-2 28935951 PMC5608957

[B40] Jayakannan M. Bose J. Babourina O. Rengel Z. Shabala S. (2015). Salicylic acid in plant salinity stress signalling and tolerance. *Plant Growth Regul.* 76 25–40. 10.1007/s10725-015-0028-z

[B41] Jini D. Joseph B. (2017). Physiological mechanism of salicylic acid for alleviation of salt stress in rice. *Rice Sci.* 24 97–108. 10.1016/j.rsci.2016.07.007

[B42] Kamiab F. Talaie A. Khezri M. Javanshah A. (2014). Exogenous application of free polyamines enhance salt tolerance of pistachio (*Pistacia vera* L.) seedlings. *Plant Growth Regul.* 72 257–268. 10.1007/s10725-013-9857-9

[B43] Kang G. Li G. Guo T. (2014). Molecular mechanism of salicylic acid-induced abiotic stress tolerance in higher plants. *Acta Physiol. Plant.* 36 2287–2297. 10.1007/s11738-014-1603-z

[B44] Kang G. Li G. Xu W. Peng X. Han Q. Zhu Y. (2012). Proteomics reveals the effects of salicylic acid on growth and tolerance to subsequent drought stress in wheat. *J. Proteome Res.* 11 6066–6079. 10.1021/pr300728y 23101459

[B45] Khan M. I. Fatma M. Per T. S. Anjum N. A. Khan N. A. (2015). Salicylic acid-induced abiotic stress tolerance and underlying mechanisms in plants. *Front. Plant Sci.* 6:462. 10.3389/fpls.2015.00462 26175738 PMC4485163

[B46] Khan M. I. R. Asgher M. Khan N. A. (2014). Alleviation of salt-induced photosynthesis and growth inhibition by salicylic acid involves glycinebetaine and ethylene in mungbean (*Vigna radiata* L.). *Plant Physiol. Biochem.* 80 67–74. 10.1016/j.plaphy.2014.03.026 24727790

[B47] Klessig D. F. Choi H. W. Dempsey D. A. (2018). Systemic acquired resistance and salicylic acid: past, present, and future. *Mol. Plant Microbe Interact.* 31 871–888. 10.1094/mpmi-03-18-0067-cr 29781762

[B48] Kohli S. K. Handa N. Kaur R. Kumar V. Khanna K. Bakshi P. (2017). “Role of salicylic acid in heavy metal stress tolerance: insight into underlying mechanism,” in *Salicylic Acid: A Multifaceted Hormone*, eds Nazar R. Iqbal N. Khan N. A. (Singapore: Springer), 123–144.

[B49] Koo Y. J. Kim M. A. Kim E. H. Song J. T. Jung C. Moon J. K. (2007). Overexpression of salicylic acid carboxyl methyltransferase reduces salicylic acid-mediated pathogen resistance in *Arabidopsis thaliana*. *Plant Mol. Biol.* 64 1–15. 10.1007/s11103-006-9123-x 17364223

[B50] Kovacik J. Gruz J. Backor M. Strnad M. Repcak M. (2009). Salicylic acid-induced changes to growth and phenolic metabolism in *Matricaria chamomilla* plants. *Plant Cell Rep.* 28 135–143. 10.1007/s00299-008-0627-5 18972114

[B51] Kumar D. Klessig D. F. (2003). High-affinity salicylic acid-binding protein 2 is required for plant innate immunity and has salicylic acid-stimulated lipase activity. *Proc. Natl. Acad. Sci. U.S.A.* 100 16101–16106. 10.1073/pnas.0307162100 14673096 PMC307699

[B52] Li C. Chen Q. Gao X. Qi B. Chen N. Xu S. (2005). AtHsfA2 modulates expression of stress responsive genes and enhances tolerance to heat and oxidative stress in *Arabidopsis. Sci. China C Life Sci.* 48 540–550. 10.1360/062005-119 16483133

[B53] Lin C. Zhang Y. X. Chai T. Y. (2008). *Arabidopsis DREB1A* confers high salinity tolerance and regulates the expression of GA dioxygenases in Tobacco. *Plant Sci.* 174 156–164. 10.1016/j.plantsci.2007.11.002

[B54] Lin J. Mazarei M. Zhao N. Hatcher C. N. Wuddineh W. A. Rudis M. (2016). Transgenic soybean overexpressing *GmSAMT1* exhibits resistance to multiple-HG types of soybean cyst nematode Heterodera glycines. *Plant Biotechnol. J.* 14 2100–2109. 10.1111/pbi.12566 27064027 PMC5095773

[B55] Lin J. Mazarei M. Zhao N. Zhu J. J. Zhuang X. Liu W. (2013). Overexpression of a soybean salicylic acid methyltransferase gene confers resistance to soybean cyst nematode. *Plant Biotechnol. J.* 11 1135–1145. 10.1111/pbi.12108 24034273

[B56] Makita Y. Gonda S. I. (2012). H2 enhances *Arabidopsis* salt tolerance by manipulating ZAT10/12-mediated antioxidant defence and controlling sodium exclusion. *PLoS One* 7:e49800. 10.1371/journal.pone.0049800 23185443 PMC3504229

[B57] Manohar M. Tian M. Moreau M. Park S. W. Choi H. W. Fei Z. (2014). Identification of multiple salicylic acid-binding proteins using two high throughput screens. *Front. Plant Sci.* 5:777. 10.3389/fpls.2014.00777 25628632 PMC4290489

[B58] Marini R. (2008). Salicylic acid may be involved in the regulation of drought-induced leaf senescence in perennials: a case study in field-grown *Salvia officinalis* L. plants. *Environ. Exp. Bot.* 64 105–112. 10.1016/j.envexpbot.2007.12.016

[B59] Mehterov N. Balazadeh S. Hille J. Toneva V. Mueller-Roeber B. Gechev T. (2012). Oxidative stress provokes distinct transcriptional responses in the stress-tolerant atr7 and stress-sensitive loh2 *Arabidopsis thaliana* mutants as revealed by multi-parallel quantitative real-time PCR analysis of ROS marker and antioxidant genes. *Plant Physiol. Biochem.* 59 20–29. 10.1016/j.plaphy.2012.05.024 22710144

[B60] Michiko Y. Atsushi I. Yusuke J. Motoaki S. Taishi U. Tadao A. (2008). Antagonistic interaction between systemic acquired resistance and the abscisic acid-mediated abiotic stress response in *Arabidopsis*. *Plant Cell* 20 1678–1692. 10.1105/tpc.107.054296 18586869 PMC2483369

[B61] Miller G. Shulaev V. Mittler R. (2008). Reactive oxygen signaling and abiotic stress. *Physiol. Plant* 133 481–489. 10.1111/j.1399-3054.2008.01090.x 18346071

[B62] Miller G. Suzuki N. Ciftci-Yilmaz S. Mittler R. (2010). Reactive oxygen species homeostasis and signalling during drought and salinity stresses. *Plant Cell Environ.* 33 453–467. 10.1111/j.1365-3040.2009.02041.x 19712065

[B63] Mittler R. (2016). ROS are good. *Trends Plant Sci.* 22 11–19. 10.1016/j.tplants.2016.08.002 27666517

[B64] Miura K. Okamoto H. Okuma E. Shiba H. Kamada H. Hasegawa P. M. (2013). SIZ1 deficiency causes reduced stomatal aperture and enhanced drought tolerance via controlling salicylic acid-induced accumulation of reactive oxygen species in *Arabidopsis*. *Plant J.* 73 91–104. 10.1111/tpj.12014 22963672

[B65] Miura K. Tada Y. (2014). Regulation of water, salinity, and cold stress responses by salicylic acid. *Front. Plant Sci.* 5:4. 10.3389/fpls.2014.00004 24478784 PMC3899523

[B66] Molders W. Buchala A. Metraux J. P. (1996). Transport of salicylic acid in tobacco necrosis virus-infected cucumber plants. *Plant Physiol. Biochem.* 112 787–792. 10.1104/pp.112.2.787 12226421 PMC158003

[B67] Mutlu S. Karadaǧoǧlu Ö. Atici Ö. Nalbantoǧlu B. (2013). Protective role of salicylic acid applied before cold stress on antioxidative system and protein patterns in barley apoplast. *Biol. Plant* 57 507–513. 10.1007/s10535-013-0322-4

[B68] Nath M. Bhatt D. Prasad R. Gill S. S. Anjum N. A. Tuteja N. (2016). Reactive oxygen species generation-scavenging and signaling during plant-arbuscular mycorrhizal and *Piriformospora indica* interaction under stress condition. *Front. Plant Sci.* 7:1574. 10.3389/fpls.2016.01574 27818671 PMC5073151

[B69] Niu C. F. Wei W. Zhou Q. Y. Tian A. G. Hao Y. J. Zhang W. K. (2012). Wheat WRKY genes *TaWRKY2* and *TaWRKY19* regulate abiotic stress tolerance in transgenic *Arabidopsis* plants. *Plant Cell Environ.* 35 1156–1170. 10.1111/j.1365-3040.2012.02480.x 22220579

[B70] Pál M. Horváth E. Janda T. Páldi E. Szalai G. (2010). Cadmium stimulates the accumulation of salicylic acid and its putative precursors in maize (*Zea mays*) plants. *Physiol. Plant.* 125 356–364. 10.1111/j.1399-3054.2005.00545.x

[B71] Panda S. K. Patra H. K. (2007). Effect of salicylic acid potentiates cadmium-induced oxidative damage in *Oryza sativa* L. leaves. *Acta Physiol. Plant.* 29 567–575. 10.1007/s11738-007-0069-7

[B72] Park S. W. Kaimoyo E. Kumar D. Mosher S. Klessig D. F. (2007). Methyl salicylate is a critical mobile signal for plant systemic acquired resistance. *Science* 318 113–116. 10.1126/science.1147113 17916738

[B73] Poque S. Wu H. W. Huang C. H. Cheng H. W. Hu W. C. Yang J. Y. (2018). Potyviral gene-silencing suppressor HCPro interacts with salicylic acid (SA)-binding protein 3 to weaken SA-mediated defense responses. *Mol. Plant Microbe Interact.* 31 86–100. 10.1094/mpmi-06-17-0128-fi 29090655

[B74] Qin Y. Tian Y. Han L. Yang X. (2013). Constitutive expression of a salinity-induced wheat WRKY transcription factor enhances salinity and ionic stress tolerance in transgenic *Arabidopsis thaliana*. *Biochem. Biophys. Res. Commun.* 441 476–481. 10.1016/j.bbrc.2013.10.088 24383079

[B75] Radhakrishnan R. Lee I. J. (2013). Spermine promotes acclimation to osmotic stress by modifying antioxidant, abscisic acid, and jasmonic acid signals in soybean. *Plant Growth Regul.* 32 22–30. 10.1007/s00344-012-9274-8

[B76] Reis R. R. da Cunha B. A. Martins P. K. Martins M. T. Alekcevetch J. C. Chalfun A. (2014). Induced over-expression of *AtDREB2A CA* improves drought tolerance in sugarcane. *Plant Sci.* 221–222, 59–68. 10.1016/j.plantsci.2014.02.003 24656336

[B77] Seskar M. Shulaev V. Raskin I. (1998). Endogenous methyl salicylate in pathogen-inoculated tobacco plants. *Plant Physiol.* 116 387–392. 10.1104/pp.116.1.387

[B78] Shafi A. Chauhan R. Gill T. Swarnkar M. K. Sreenivasulu Y. Kumar S. (2015). Expression of *SOD* and *APX* genes positively regulates secondary cell wall biosynthesis and promotes plant growth and yield in *Arabidopsis* under salt stress. *Plant Mol. Biol.* 87 615–631. 10.1007/s11103-015-0301-6 25754733

[B79] Shakirova F. M. Sakhabutdinova A. R. Bezrukova M. V. Fatkhutdinova R. A. Fatkhutdinova D. R. (2003). Changes in the hormonal status of wheat seedlings induced by salicylic acid and salinity. *Plant Sci.* 164 317–322. 10.1016/S0168-9452(02)00415-6

[B80] Shi W. Hao L. Li J. Liu D. Guo X. Li H. (2014). The *Gossypium hirsutum* WRKY gene *GhWRKY39-1* promotes pathogen infection defense responses and mediates salt stress tolerance in transgenic *Nicotiana benthamiana*. *Plant Cell Rep.* 33 483–498. 10.1007/s00299-013-1548-5 24337818

[B81] Siegrist J. Orober M. Buchenauer H. (2000). β-Aminobutyric acid-mediated enhancement of resistance in tobacco to tobacco mosaic virus depends on the accumulation of salicylic acid. *Physiol. Mol. Plant Pathol.* 56 95–106. 10.1006/pmpp.1999.0255

[B82] Slaymaker D. H. Navarre D. A. Clark D. del Pozo O. Martin G. B. Klessig D. F. (2002). The tobacco salicylic acid-binding protein 3 (SABP3) is the chloroplast carbonic anhydrase, which exhibits antioxidant activity and plays a role in the hypersensitive defense response. *Proc. Natl. Acad. Sci. U.S.A.* 99 11640–11645. 10.1073/pnas.182427699 12185253 PMC129322

[B83] Song J. T. Koo Y. J. Park J. B. Seo Y. J. Cho Y. J. Seo H. S. (2009). The expression patterns of *AtBSMT1* and *AtSAGT1* encoding a salicylic acid (SA) methyltransferase and a SA glucosyltransferase, respectively, in *Arabidopsis* plants with altered defense responses. *Mol. Cells* 28 105–109. 10.1007/s10059-009-0108-x 19669626

[B84] Song J. T. Koo Y. J. Seo H. S. Kim M. C. Choi Y. D. Kim J. H. (2008). Overexpression of AtSGT1, an *Arabidopsis* salicylic acid glucosyltransferase, leads to increased susceptibility to *Pseudomonas syringae*. *Phytochemistry* 69 1128–1134. 10.1016/j.phytochem.2007.12.010 18226820

[B85] Sreenivasulu N. Sopory S. K. Kavi Kishor P. B. (2007). Deciphering the regulatory mechanisms of abiotic stress tolerance in plants by genomic approaches. *Gene* 388 1–13. 10.1016/j.gene.2006.10.009 17134853

[B86] Strawn M. A. Marr S. K. Inoue K. Inada N. Zubieta C. Wildermuth M. C. (2007). *Arabidopsis* isochorismate synthase functional in pathogen-induced salicylate biosynthesis exhibits properties consistent with a role in diverse stress responses. *J. Biol. Chem.* 282 5919–5933. 10.1074/jbc.M605193200 17190832

[B87] Sun J. Hu W. Zhou R. Wang L. Wang X. Wang Q. (2015). The brachypodium distachyon *BdWRKY36* gene confers tolerance to drought stress in transgenic tobacco plants. *Plant Cell Rep.* 34 23–35. 10.1007/s00299-014-1684-6 25224555

[B88] Szepesi A. Csiszar J. Gemes K. Horvath E. Horvath F. Simon M. L. (2009). Salicylic acid improves acclimation to salt stress by stimulating abscisic aldehyde oxidase activity and abscisic acid accumulation, and increases Na+ content in leaves without toxicity symptoms in *Solanum lycopersicum* L. *J. Plant Physiol.* 166 914–925. 10.1016/j.jplph.2008.11.012 19185387

[B89] Thomas W. T. (2015). Drought-resistant cereals: impact on water sustainability and nutritional quality. *Proc. Nutr. Soc.* 74 191–197. 10.1017/s0029665115000026 25702698

[B90] Tripathi D. Jiang Y. L. Kumar D. (2010). SABP2, a methyl salicylate esterase is required for the systemic acquired resistance induced by acibenzolar-S-methyl in plants. *FEBS Lett.* 584 3458–3463. 10.1016/j.febslet.2010.06.046 20621100

[B91] Vlot A. C. Dempsey D. A. Klessig D. F. (2009). Salicylic acid, a multifaceted hormone to combat disease. *Annu. Rev. Phytopathol.* 47 177–206. 10.1146/annurev.phyto.050908.135202 19400653

[B92] Vlot A. C. Liu P. P. Cameron R. K. Park S. W. Yang Y. Kumar D. (2008). Identification of likely orthologs of tobacco salicylic acid-binding protein 2 and their role in systemic acquired resistance in *Arabidopsis thaliana*. *Plant J.* 56 445–456. 10.1111/j.1365-313X.2008.03618.x 18643994

[B93] Wahid A. (2009). Advances in drought resistance of rice. *Crit. Rev. Plant Sci.* 28 199–217. 10.1080/07352680902952173

[B94] Wang Y. Branicky R. Noe A. Hekimi S. (2018). Superoxide dismutases: dual roles in controlling ROS damage and regulating ROS signaling. *J. Cell Biol.* 217 1915–1928. 10.1083/jcb.201708007 29669742 PMC5987716

[B95] Wang Y. Q. Feechan A. Yun B. W. Shafiei R. Hofmann A. Taylor P. (2009). S-nitrosylation of AtSABP3 antagonizes the expression of plant immunity. *J. Biol. Chem.* 284 2131–2137. 10.1074/jbc.M806782200 19017644

[B96] Wei Q. Luo Q. Wang R. Zhang F. He Y. Zhang Y. (2017). A wheat R2R3-type MYB transcription factor TaODORANT1 positively regulates drought and salt stress responses in transgenic tobacco plants. *Front. Plant Sci.* 8:1374. 10.3389/fpls.2017.01374 28848578 PMC5550715

[B97] Wei T. Deng K. Zhang Q. Gao Y. Liu Y. Yang M. (2017). Modulating *AtDREB1C* expression improves drought tolerance in *Salvia miltiorrhiza*. *Front. Plant Sci.* 8:52. 10.3389/fpls.2017.00052 28174590 PMC5259653

[B98] Wei T. Deng K. Liu D. Gao Y. Liu Y. Yang M. (2016). Ectopic expression of DREB transcription factor, AtDREB1A, confers tolerance to drought in transgenic *Salvia miltiorrhiza*. *Plant Cell Physiol.* 57 1593–1609. 10.1093/pcp/pcw084 27485523

[B99] Wu D. Ji J. Wang G. Guan C. Jin C. (2014). LchERF, a novel ethylene-responsive transcription factor from *Lycium chinense*, confers salt tolerance in transgenic tobacco. *Plant Cell Rep.* 33 2033–2045. 10.1007/s00299-014-1678-4 25182480

[B100] Xia X. J. Zhou Y. H. Shi K. Zhou J. Foyer C. H. Yu J. Q. (2015). Interplay between reactive oxygen species and hormones in the control of plant development and stress tolerance. *J. Exp. Bot.* 66 2839–2856. 10.1093/jxb/erv089 25788732

[B101] Xiao-Yun L. Xu L. Yao Y. Yi-Hao L. Shuai L. Chao-Yong H. (2013). Overexpression of *Arachis hypogaea AREB1* gene enhances drought tolerance by modulating ROS scavenging and maintaining endogenous ABA content. *Int. J. Mol. Sci.* 14 12827–12842. 10.3390/ijms140612827 23783278 PMC3709814

[B102] Xu Q. Xu X. Zhao Y. Jiao K. Herbert S. J. Hao L. (2008). Salicylic acid, hydrogen peroxide and calcium-induced saline tolerance associated with endogenous hydrogen peroxide homeostasis in naked oat seedlings. *Plant Growth Regul.* 54 249–259. 10.1007/s10725-007-9247-2

[B103] You J. Chan Z. (2015). ROS regulation during abiotic stress responses in crop plants. *Front. Plant Sci.* 6:1092. 10.3389/fpls.2015.01092 26697045 PMC4672674

[B104] Zhang F. Zhang H. Xia Y. Wang G. Xu L. Shen Z. (2011). Exogenous application of salicylic acid alleviates cadmium toxicity and reduces hydrogen peroxide accumulation in root apoplasts of *Phaseolus aureus* and *Vicia sativa*. *Plant Cell Rep.* 30 1475–1483. 10.1007/s00299-011-1056-4 21409549

[B105] Zhang Y. Xu S. Yang S. Chen Y. (2015). Salicylic acid alleviates cadmium-induced inhibition of growth and photosynthesis through upregulating antioxidant defense system in two melon cultivars (*Cucumis melo* L.). *Protoplasma* 252 911–924. 10.1007/s00709-014-0732-y 25398649

[B106] Zheng X. Li Q. Liu D. Zang L. Zhang K. Deng K. (2015). Promoter analysis of the sweet potato ADP-glucose pyrophosphorylase gene *IbAGP1* in *Nicotiana tabacum*. *Plant Cell Rep.* 34 1873–1884. 10.1007/s00299-015-1834-5 26183951

[B107] Zhu M. Monroe J. G. Suhail Y. Villiers F. Mullen J. Pater D. (2016). Molecular and systems approaches towards drought-tolerant canola crops. *New Phytol.* 210 1169–1189. 10.1111/nph.13866 26879345

